# Anti-SARS-CoV-2 VHH and C7C peptide fused with Angiopep-2 efficiently traverse blood-brain barrier model and neutralizes virus

**DOI:** 10.3389/fmicb.2026.1827887

**Published:** 2026-06-18

**Authors:** Mangesh Bhide, Katarina Bhide, Evelina Mochnacova, Jakub Viglasky, Diana Kosturikova, Amod Kulkarni

**Affiliations:** 1 Laboratory of Biomedical Microbiology and Immunology, University of Veterinary Medicine and Pharmacy in Košice, Košice, Slovakia; 2 Institute of Neuroimmunology of Slovak Academy of Sciences, Bratislava, Slovakia

**Keywords:** Angiopep-2, blood-brain barrier, C7C peptide, combinatorial phage display, SARS-CoV-2, VHH, VHH-phage display, virus neutralization

## Abstract

**Introduction:**

The therapy for SARS-CoV-2-related CNS infection relies on treating neuroinflammation and associated damage, rather than a specific antiviral. The lack of specific antiviral therapeutics is mainly attributed to the limited permeability of drugs across the blood-brain barrier.

**Methods:**

In this paper, we present a detailed pipeline for selecting VHHs (single variable domain of llama heavy chain antibody) and 7-mer cyclic peptides (C7C) from phage-display libraries that bind to the spike protein and SARS-CoV-2 virion. Soluble VHH and C7C peptides overexpressed in the *E. coli* SHuffle Express, which enables the formation of the disulfide bond essential for proper VHH and C7C folding and function, were purified and rigorously tested for their capacity to neutralize pseudovirus, cell toxicity, hemocompatibility, etc. The best VHH and C7C candidates with favorable attributes were then fused with the CNS-homing peptide Angiopep-2 and thoroughly assessed for the ability to neutralize the virus along with other safety attributes mentioned earlier.

**Results and discussion:**

The best VHH and C7C candidates, VHH_*E12*_ and C7C_18_, tested for their ability to neutralize live virus in a plaque reduction neutralization test, showed EC_50_ 0.045 μg/mL and 0.01 μg/mL, respectively. Both VHH_*E12*_ and C7C_18_, however, fail to cross the blood-brain barrier in vitro, thus were fused with CNS-homing peptide Angiopep-2. VHH_*E12*_-Angiopep-2 and C7C_18_-Angiopep-2 fusion constructs showed increased crossing across the blood-brain barrier by 5.4-fold and 11.2-fold, respectively, while maintaining their ability to neutralize the virus. Molecules generated here, particularly C7C_18_-Angiopep-2, hold merit for further in vivo testing and preclinical studies.

## 1 Introduction

Over the past decade, members of the Coronaviridae family, including SARS-CoV (2002), Middle East respiratory syndrome coronavirus (MERS-CoV, 2012), and SARS-CoV-2 (COVID-19, 2019), have caused severe respiratory diseases in humans and animals. While earlier coronaviruses exhibited high case fatality rates (approximately 9.6% for SARS-CoV and 34% for MERS-CoV), their overall spread remained relatively limited (Zumla et al., 2015). On the other hand, SARS-CoV-2 has proven exceptionally difficult to contain due to its high transmissibility, resulting in approximately 778 million confirmed cases and 7.1 million deaths worldwide by December 2025 (WHO, 2020). Beyond respiratory disease, SARS-CoV-2 exhibits broad tissue tropism, with documented infection of the heart, kidneys, liver, spleen, and the central nervous system (Lopes-Pacheco et al., 2021). Unlike previous coronaviruses, SARS-CoV-2 uniquely combines high transmissibility with multi-organ involvement, including invasion of the central nervous system (CNS).

The neurological symptoms of SARS-CoV-2 infection have changed as the virus evolved and new variants emerged. At the outset of the COVID-19 pandemic, loss of smell and taste was identified as a common symptom of infection; however, encephalitis and several associated neurological symptoms, such as headaches, nausea, dizziness, and often neurocognitive symptoms (the brain fog), were reported. Viral particles of SARS-CoV-2 have been discovered in the brain, both in the cerebrospinal fluid (CSF) and in the gray matter (Fisicaro et al., 2021; Pajo et al., 2021; Thakur et al., 2021), while neuropathological features are mainly associated with the Delta and Omicron variants (Normandin et al., 2023). It was recently shown that SARS-CoV-2 has tropism mainly for human cortical astrocytes (Andrews et al., 2022), and both Delta and Omicron induce several signaling pathways in the astrocytes that are associated with brain fog and long COVID (Bhide et al., 2025). Despite the plethora of research being accumulated, there are scanty reports available on the development of antiviral molecules that are capable of crossing the blood-brain barrier (BBB) and neutralizing the neuroinfection. Typically, only a very small fraction, around 0.1%–0.2%, of large molecules like therapeutic antibodies can cross the BBB into the central nervous system from the bloodstream, significantly limiting their effectiveness (Bajracharya et al., 2021). As a result, the development of effective therapeutic molecules that can cross the BBB is highly desirable.

Severe acute respiratory syndrome coronavirus-2 consists of four structural proteins, namely, spike protein, small envelope glycoprotein, membrane glycoprotein, and nucleocapsid protein (Jiang et al., 2020). The spike protein helps the viral attachment to the angiotensin-converting enzyme 2 (ACE2) receptor on the host cell, which initiates viral entry into the cell. Spike glycoprotein is composed of two subunits, the N-terminal S1 domain (which contains the receptor-binding domain - RBD) and the membrane-bound C-terminal S2 region (which contains the fusion peptide and two heptad-repeat domains). As the RBD is involved in receptor attachment, it is the key molecule in the development of viral cell-entry blocking therapeutics (Dong Y. et al., 2020; Dai and Gao, 2021; Malik et al., 2021). Several molecules, including monoclonal antibodies and single-chain variable fragments (scFvs), are being developed for therapeutic applications that target the spike protein (Cao et al., 2020; Tian et al., 2020; Bertoglio et al., 2021; Weinreich et al., 2021); however, they are costly and cumbersome to produce. Among other molecules, VHHs (also known as nanobodies) and small peptides (e.g., phage-display-derived 7- or 12-mer) can overcome some of the pitfalls of conventional monoclonal antibodies, as they can be produced cost-effectively on a large scale in recombinant protein expression systems (such as *E. coli* or *Pichia pastoris*) or chemical synthesis. Another advantage of nanobodies and peptides is their small size, which is typically 10–20 times smaller than conventional IgG, allowing improved biodistribution even in niches such as joints and the brain parenchyma (Iwasaki and Yang, 2020).

Several combinatorial phage libraries displaying 7–12-mer linear peptides or structurally constrained cyclic peptides (e.g., 7-mer cyclic peptide display library offered by New England Biolabs) have been used by us and others for the development of antimicrobial and antiviral molecules (Rajik et al., 2009; Mertinkova et al., 2021; Sokullu et al., 2021; Mochnacova et al., 2025; Petrouskova et al., 2025). Cyclic peptides, in particular, offer several advantages over linear peptides, primarily due to their rigid, ring-shaped structure. Cyclization often generates a more compact and globular structure, enabling the peptide to engage more efficiently with its target. Several studies imply that cyclic peptides have lower minimum inhibitory concentrations (MIC) than their linear counterparts (Oh et al., 2014; Mitra et al., 2025). They provide higher stability against metabolic degradation, higher binding affinity and selectivity for targets, improved bioavailability, and cell membrane permeability (Martian et al., 2025). These characteristics make cyclic peptides perfect candidates for therapeutic drug development. Some known naturally occurring antimicrobials that contain cyclic peptides or motifs include vancomycin, daptomycin, and clovibactin (Shriwastav et al., 2025). When developing a molecule to bind the RBD and block its interaction with the cell receptor, it is ideal for the blocking molecule to have a rigid structure and be able to perfectly match the surface of the viral protein (such as SARS-CoV-2 spike protein) in order to minimize off-target effects. We recently developed cyclic peptides against protein E of West Nile virus and tick-borne encephalitis virus, which demonstrated enhanced viral blocking ability compared to the linear peptides derived from the 12-mer combinatorial phage library (Mertinkova et al., 2021; Petrouskova et al., 2025). A cyclic peptide with promising activity against the protease of SARS-CoV-2 was also reported recently (Johansen-Leete et al., 2022). Thus, in the present work, we employed a phage library displaying cyclic peptides, structurally constrained by two cysteine residues at both ends of a chain of seven amino acids (C7C phage display peptide library).

Several studies have reported modification of biomolecules to increase their translocation across the BBB via receptor-mediated endocytosis (Regina et al., 2015; Guo et al., 2020; Majerova et al., 2020). Nanobodies and peptides have favorable biophysical features, as they can be easily fused with the CNS-homing ligands for increased BBB transport. When fused with a CNS-homing ligand, therapeutically relevant concentrations of the virus-neutralizing moiety can be achieved within the brain parenchyma. For instance, the Angiopep-2 (a CNS homing ligand) covalently linked to monoclonal antibodies showed significantly improved translocation across the BBB (Regina et al., 2015). The TGN peptide (another CNS homing ligand) fused to nanoparticles carrying neuroprotective peptides resulted in enhanced neuronal delivery (Guo et al., 2020). In the recent past, several homing peptides of the 1st, 2nd, and 3rd generations have been developed (Diaz-Perlas et al., 2018); however, Angiopep-2 remains one of the most effective homing peptides for drug delivery, which overcomes the BBB without disrupting it, and is currently the most advanced BBB shuttle in clinical trials (Dmello et al., 2024). Its primary advantage lies in exploiting receptor-mediated transcytosis (RMT) by targeting the low-density lipoprotein receptor-related protein-1 (LRP1), which is highly expressed on the BBB (Habib and Singh, 2022). Angiopep-2 has demonstrated higher transcytosis capacity compared to aprotinin in *in vitro* BBB models. It also outperforms transferrin receptor (TfR) ligands (like TfRL) and other brain-shuttle peptides in parenchyma infiltration (Habib and Singh, 2022). It is easily conjugated to various nanocarriers (such as liposomes and PAMAM dendrimers) and antibodies while retaining its binding affinity, demonstrating its translational potential (Regina et al., 2015; Kumthekar et al., 2020; Habib and Singh, 2022; Jozefiakova et al., 2025). Together, these advances illustrate the potential of the fusion of a homing peptide, particularly Angiopep-2, to VHH or C7C peptides as an early proof-of-concept molecule for optimizing both virus neutralization and BBB crossing.

In this paper, we describe an experimental pipeline that begins with the selection of VHH and C7C peptides against the RBD of spike protein using two distinct phage libraries (the nanobody-phage and C7C combinatorial phage library). This is followed by the selection of VHH and C7C clones based on their affinity to the virus, ability to neutralize the two pseudovirus strains as well as the live Omicron variant, and non-cytotoxicity. The best VHH and C7C candidates were subsequently fused with Angiopep-2 and tested for their ability to cross the BBB *in vitro* and neutralize the virus. This experimental approach successfully produced a VHH_*E12*_, and C7C_18_, which were able to neutralize the virus with EC_50_ 0.045 μg/mL and 0.01 μg/mL, respectively. Both candidates did not show any hemolytic activity or cell toxicity, however they were unable to efficiently cross the blood-brain barrier *in vitro*. The fusion of VHH_*E12*_ and C7C_18_ with Angiopep-2 resulted in enhanced BBB traversal, particularly for C7C_18_-Angiopep-2, which was 11.2-fold more than C7C_18_. Both VHH_*E12*_ and C7C_18_ retained their ability to neutralize the virus after fusion with Angiopep-2. The molecules developed here possess the potential of being developed further into efficient therapeutics against the neuroinfection caused by SARS-CoV-2.

## 2 Materials and methods

### 2.1 Spike protein and its receptor binding domain (RBD)

Recombinant forms of the spike protein and RBD were produced in Expi293 expression system (cells - Expi293F cells, ThermoFisher Scientific, USA, cat. Number A14635), purified, and checked for purity with SDS-PAGE and MALDI mass spectrometry. Details are provided in Supplementary information 1.

### 2.2 VHH-phage library and panning for selection of phage clones having affinity to spike protein

Spike protein was employed for *in vitro* immunization of llama lymphocytes exactly as described in our previous publication (Comor et al., 2017). Following immunization, the RNA was extracted, reverse transcribed, and the VHH coding sequence was amplified, ligated into the pjB12 phagemid and electroporated into *E. coli*. The phages expressing nanobodies on their pIII protein were packaged with VCSM13 interface-resistant helper phage and escaped from *E. coli* (designated as the VHH-phage library). Supplementary information 2 provides details of library preparation. The library was used in the panning.

First, to eliminate nonspecific interactions, the VHH-phage library (3.9 × 10^11^ phages) was preincubated for 1 h at 37 °C in a nickel-coated plate and then an albumin-coated plate to deplete non-specific VHHs that bind to nickel, plastic or albumin. The preincubated library was then allowed to bind to recombinant RBD immobilized on a nickel plate to select VHH clones having affinity to RBD. Unbound phages were washed, and the phage-RBD complex was eluted using imidazole. Eluted phages were amplified in *E. coli* culture and then purified using PEG-NaCl. Purified phages were enumerated and used in the second round of panning against RBD. A total of three rounds of panning were performed. A detailed experimental procedure is provided in the Supplementary information 2.

### 2.3 C7C-phage library and selection of clones having affinity to spike protein

The combinatorial phage display was employed to isolate C7C peptides. The Ph.D.-C7C library (New England Biolabs, USA) was panned in three rounds of selection. A detailed procedure is described in Supplementary information 3. Following the final round of panning, phages were amplified, precipitated, and stored at −80 °C as a glycerol stock.

### 2.4 Isolation of individual VHH-phage and C7C-phage clones, and assessment of their binding to spike protein

First, we used phage ELISA to determine whether the phages from the third panning (phage pool) could bind the spike protein (details are provided in Supplementary information 4). After validating the binding, the phage pools were subjected to serial dilution. In the case of VHH-phages, the dilutions were incubated with *E. coli* XL-1 and then spread on 2xTY agar plate containing antibiotics (details are provided in Supplementary information 5). In case of C7C-phages, serially diluted phase from 3rd elution were incubated with *E. coli* ER2738 (New England Biolabs, USA) for 1 h and then spread on the 2xTY agar plates (Supplementary information 5). After an overnight incubation, well-separated colonies (in case of VHH-phages) or plaques (in the case of C7C-phages) were picked and propagated in small scale (Supplementary information 5). Phages released in the supernatant were precipitated and evaluated for its ability to bind to spike protein using phage ELISA (Supplementary information 6).

### 2.5 Sequencing of the phage clones that show affinity to spike protein

Phage clones that showed affinity to spike protein (OD > 0.8 in phage ELISA) were subjected to DNA sequencing to identify VHH or C7C sequences. Supplementary information 7 provides details of DNA isolation from phage clones, PCR amplification with phagemid-specific primers, and DNA sequencing. The amino acid sequences (*in silico* translated DNA sequences) of each clone were aligned *in silico* and clustered by sequence homology (Geneious Pro 9.1, Biomatters, USA).

### 2.6 Production of soluble VHHs and C7C

Representative phage clones from each cluster were used to amplify the VHH or C7C sequence, and the amplicons were ligated in the expression vector to produce a soluble form of protein. Both VHH and C7C were overexpressed in the *E. coli* Shuffle Express (New England Biolabs, USA), which allows the formation of disulfide bonds. The VHH was tagged with an N-terminal His tag. All C7C peptides were flanked with the GGGS sequence at the C-terminus (CX_7_CGGGS) as suggested by the manufacturer of Ph.D. libraries (New England Biolabs). C7C peptides were also tagged with an N-terminal His tag. PCR conditions, digestion, and ligation into the expression vector, *E. coli* transformation, clonal selection, protein overexpression, purification, and quality control are presented in Supplementary information 8 for both VHH and C7C.

Purified VHH and C7C were evaluated for their ability to bind to virions in ELISA. In brief, wells containing purified virions (10^5^ PFU) of the Omicron variant (lineage BA.5, kindly provided by the Robert Koch Institute through the European Virus Archive Global; virions fixed with 4% paraformaldehyde) were blocked with 5% BSA in PBST (PBS containing 0.05% Tween 20, pH 7.2) for 1 h. One μg of each VHH and C7C peptide (having an N-terminal His tag) diluted in 100 μl of PBST was incubated with virions for 1 h and wells were washed 3 times with PBST. Nickel-HRP probe (diluted 1:5000 in PBST, ThermoFisher Scientific) was added to each well and incubated for 1 h. Wells were washed, and a chromogenic reaction was developed in 25 min by 1-Step Ultra TMB substrate (Thermo Fisher Scientific). The reaction was stopped with 2 M H_2_SO_4_, and the absorbance was measured at 450 nm (Multilabel plate reader, Parkin Elmer). For background control, the virions were excluded from the assay. The absorbance from the background control was used to normalize the reading from the assay. For the input control, wells coated with each VHH and C7C were incubated with the nickel-HRP probe. Another input control was performed to confirm virion attachment in the well. For that, three wells coated with virions were incubated with anti-SARS-CoV-2 HRP antibody (1:10 000 in PBS, Sino Biologicals, China), and the chromogenic reaction was developed with Ultra TMB. The assay was performed in triplicate.

### 2.7 SARS-CoV-2 pseudovirus (virus-like particle, VLP) neutralization by VHH or C7C

Two different SARS-CoV-2 pseudoviruses were used in the assay, viz., (1) replication-deficient MLV pseudotyped with SARS-CoV-2 spike protein carrying the original D614 genotype (GenBank Accession YP_009724390.1). (2) replication-deficient MLV pseudotyped with SARS-CoV-2 spike protein carrying the delta variant B.1.617 (G/452R.V3; GISAID sequence number EPI_ISL_1547802, Creative Diagnostics, UK).

For the pseudovirus neutralization assay, 1 μg of soluble VHHs or C7C was serially diluted and incubated with VLP (400–500 TCID_50_/ml; calculation of TCID_50_ is in Supplementary information 9) for 90 min at room temperature. Details of dilution and various controls used in the neutralization assay are also in Supplementary information 9. The preincubated mix of VHH-pseudovirus or C7C-pseudovirus was transferred to overnight-grown HEK293/17 cells (Name – 293T/17 [HEK 293T/17], Source - human embryo kidney tissue, ATCC, USA, cat. Number CRL-11268) and incubated for 48 h at 37 °C in 5% CO2. After incubation, the supernatant was removed carefully, and 20 μl lysis buffer provided in the luciferase assay system kit (Promega, USA) was added to each well. Cells were lysed for 5 min at room temperature, and the lysate was transferred to a 96-well plate (white-walled and bottom wells; CELLSTAR, USA), and 100 μl of the luciferase assay reagent was added before measuring the luminescence on Cytation 7 (Biotek, USA) with the parameters: integration time: 10 s, read height: 5.4 mm and gain: 240. The amount of VLP entering the target cells was calculated by detecting the expression of luciferase, which was then used to measure the neutralizing ability of the VHH or C7C, expressed in half maximal effective concentration (EC_50_). To calculate EC_50_, values obtained from Cytation 7 were pasted in the Excel template published in our previous publication (Hruskovicova et al., 2022). EC_50_ values were used to calculate half maximal effective concentration (nanograms) of VHH/C7C required to neutralize the virus using nonlinear regression (curve fit, Prism 8, Graph Pad, USA^1^). A curve was plotted using the dilution of nanobody on the *Y* axis and the amount of VHH or C7C/well on the *X* axis exactly as described by us in the previous report (Hruskovicova et al., 2022). To interpolate unknowns from the standard curve, a sigmoidal (sigmoid, 4PL, x is concentration) model was used in Prism 8.

### 2.8 Assessment of the hemocompatibility of VHH and C7C

In brief, 30 mL of heparinized sheep blood (purchased from Krigo s.r.o., Slovakia) was centrifuged at 2000 RPM for 5 min to separate erythrocytes, and 3 washings with 0.9% saline were performed. Washed erythrocytes were resuspended in 150 mL of 0.9% saline, and 1 mL of erythrocyte suspension was incubated with 1 mL of the VHH or C7C with the final concentration adjusted to 10 μg for 5 h at 37 °C. Similarly, 1 mL of erythrocyte suspension was mixed with either 1 mL of 2% Triton X 100 (Sigma) or 0.9% saline to serve as positive and negative controls, respectively. After the termination of incubation periods, all the test and control samples were centrifuged (2000 RPM for 5 min), and the supernatant containing hemoglobin was allowed to oxidize at room temperature for 30 min. The absorbance of oxyhemoglobin in all the samples (1 mL) was measured at 414 nm (NanoDrop, Thermo Fisher Scientific). Hemolysis in erythrocytes was calculated using the formula: % hemolysis = (absorbance_*sample*_ − absorbance_negative control_)/(absorbance_positive control_ − absorbance_negative control_) × 100. The entire assay was performed in duplicate.

### 2.9 Assessment of the cytotoxicity caused by VHH or C7C

The VHH and C7C peptides were examined for their potential cytotoxicity on HEK293/17 cells using the Cell Proliferation Assay Kit (XTT, AppliChem). Briefly, HEK293/17 cells (Passage 4, 20,000 cells per well) were grown in a 96-well plate containing 100 μL of DMEM high glucose complete medium (Merck, USA) until they attain 70% confluence. Thereafter, 2 μg of VHHs or C7C diluted into 100 μl of DMEM high-glucose complete medium was added. For positive control, cells were incubated with 0.01% Triton X 100, whereas the cells incubated with PBS served as negative control. Wells devoid of cells but containing DMEM high-glucose complete medium were maintained to use them for blank reading. After the completion of 24 h incubation at 37 °C in a 5% CO_2_, all the wells were added with 50 μl of XTT reagent, and the incubation was continued for 3 more hours. Absorbance in all the wells was measured at 450 nm in an ELISA plate reader, and the viability of cells treated with VHH or C7C peptides was determined using the formula (absorbance_*sample*_ − Mean absorbance_*blank*_)/(Mean absorbance_*negative control*_ − Mean absorbance_*blank*_) × 100. The assay was performed in triplicate.

### 2.10 Plaque reduction neutralization test (PRNT)

Severe acute respiratory syndrome coronavirus-2 (lineage BA.5) was propagated in VERO E6 cells (name VERO C1008 clone E6, source - African green monkey kidney cells, ATCC, USA, cat number C-1008), titrated, and used in a plaque reduction neutralization test to assess the neutralizing activity of VHH and C7C at different concentrations. Experimental details of virus titration, dilution of VHH and C7C, and PRNT are provided in Supplementary information 10.

### 2.11 VHH_*E12*_-Angiopep-2 and C7_*C*18_-Angiopep-2

The VHH_*E12*_ and C7C_18_ fused with Angiopep-2 were prepared to increase their traversal across the blood-brain barrier.

The strategy to fuse the Angiopep-2 is described in Supplementary information 11. In case of VHH-Angiopep-2 an expression cassette was prepared in which a 6xHis tag was followed by sequence coding Angiopep-2 and VHH_*E12*_. Details of the primers used to amplify the insert, vector, electroporation into *E. coli* Shuffle and protein purification are presented in Supplementary information 11. Purified VHH_*E12*_-Angiopep-2 was resolved on SDS-PAGE to check protein purity.

The C7C_18_ and C7C_18_-Angiopep-2 were procured commercially (MedChemExpress, USA). All molecules were tested for cell toxicity and hemocompatibility as described above. All molecules were conjugated with infrared dye (IRDye 680LT peptide labeling reagent, Licor bio, USA). Details are in Supplementary information 11. Molecular weights of fused and unfused molecules are confirmed on MALD-TOF as described in Supplementary information 1.

### 2.12 Biolayer interferometry for assessment of binding of Angiopep-2 fused C7C_18_ and VHH_*E12*_ to RBD

The affinity of molecules was measured using the Fortebio BLItz system (Fortebio, USA). Streptavidin-coated biosensors (SAX 2 sensors, ForteBio, USA) were hydrated for 10 min in PBS containing Tween 20 (PBST) and then dipped in biotinylated recombinant RBD (15 μg/ml) for 120 s with a shaker speed of 2200 RPM, followed by washing for 120 s with a shaker speed of 2200 RPM. PBST was used as a sample buffer and kinetics buffer. The sensor was washed with PBST for 30 s, and the affinity of C7C18 and VHHE12 (65 pmol) was measured for 240 s (120 s association and 120 s dissociation step). The total run settings were: 30 s for baseline, followed by RBD loading for 120 s, washing for 30 s, association for 120 s, and dissociation for 120 s (all steps with shaker speed of 2200 RPM). PBS was used as a control and used for normalization. Data was analyzed in BLItz Pro Software (Fortebio) and visualized in GraphPad Prism.

### 2.13 Virus neutralization by VHH_*E12*_-Angiopep-2 and C7_*C18*_-Angiopep-2

We tested if the conjugation of VHH_*E12*_ and C7C_18_ with Angiopepe-2 causes a loss of neutralization ability. VHH_*E12*_ and C7C_18_ conjugated with or without Angiopep-2 (EC_50_ concentration) were preincubated with live virus (lineage BA.5, Omicron) for 90 min and then allowed to infect the VERO E6 cells. The inhibition of the virus was assessed against virus control (no preincubation with test compounds). Preincubation of the virus with hyperimmune serum served as a control reaction, while cells without any infection or test compounds served as cell control. Experimental details are provided in Supplementary information 12. The assay was performed in triplicate.

Both Angiopep-2 conjugates were tested for hemocompatibility and their effect on cell metabolic activity exactly as described above (hemocompatibility assay and XTT). The XTT was performed on different cell types viz. HEK293/17, VERO E6, brain microvascular endothelial cells (BMEC-5i, ATCC, CRL-3245, source – human brain, cerebral cortex), and pericytes (human brain vascular pericytes, Sciencell, cat number – 1200, source – brain cortex). BMEC5i were cultured in EndoGRO-MV complete culture medium (Sigma Aldrich, USA), and pericytes were cultured in Pericyte Medium (Sciencell) as per manufacturers’ instructions till 70%–80% confluence and then used for the XTT assay.

### 2.14 Crossing of VHH_*E12*_-Angiopep-2 and C7_*C18*_-Angiopep-2 across BBB

Both Angiopep-2 fusion molecules were tested for their ability to cross the BBB *in vitro*. The BBB model was cultured on 24-transwell inserts (1 μm pores; cellQuart, Germany, transwell system) using human brain microvascular endothelial cells (Name – hCMEC D3 cell line, Merck Millipore, USA, cat number SCC066). Details of the culture of the BBB model are described in detail in Supplementary information 13. After confirming the quality of the barrier with transendothelial electrical resistance (TEER) and dextran permeability, 65 pmol of each test compound was added to the luminal chamber of the transwell and allowed to cross through the barrier to the abluminal compartment. Time-dependent (1, 3, and 5 h post-inoculation) measurement was performed using the infrared Odyssey CLx imaging system (Li-cor bio, USA). Details of the translocation assay are presented in Supplementary information 13. The assay was performed in triplicate.

### 2.15 Mapping of a plausible binding sites for VHH_*E12*_ and C7_*C18*_ on spike RBD

Mapping of the binding sites was performed as described previously (Mertinková et al., 2020; Víglaský et al., 2026). In short, 5 μg of recombinant VHH_*E12*_ or C7_*C18*_ was spotted onto an activated Immobilon-FL PVDF membrane (Millipore, USA) and air-dried overnight. The membrane was washed twice in tris-buffered saline (TBS, pH 7.2) and then incubated in TBS containing 10 μg of recombinant RBD for 2 h at room temperature with gentle agitation. Following incubation, the membrane was washed six times with TBS (1 min per wash) and air-dried overnight prior to trypsin digestion. Limited trypsin digestion of the formed complexes was carried out using 1 μg of Trypsin Gold (Promega, USA) in 300 μl of pre-warmed (37 °C) ammonium bicarbonate buffer (20 mM, pH 7.6) for 1 h. The membrane was subsequently washed six times with ammonium bicarbonate buffer to remove peptides of RBD that are not bound to VHH_*E1*_2 or C7C_18_. To elute bound peptides, the membrane was vortexed for 1 min in 10 μl of ∼98% formic acid (Sigma-Aldrich), followed by the addition of 50 μl of ≥99.9% acetonitrile (Sigma-Aldrich). After a second vortexing step, the supernatant was collected. Samples were vacuum-dried at 60 °C, and 5 μl of TA5 (acetonitrile 5%: 0.1% trifluoroacetic acid in water 95%, Sigma-Aldrich) was added. Peptides were concentrated/purified using ZipTip C18 (Millipore) according to the manufacturer’s instructions and eluted in α-cyano-4-hydroxycinnamic acid (HCCA; Bruker Daltonics, Germany) matrix prepared in TA50. One microliter of the eluate was spotted onto an AnchorChip target plate (Bruker Daltonics) and air-dried. Mass spectra were acquired using a MALDI-TOF Microflex LRF mass spectrometer (Bruker Daltonics) in reflectron-positive mode at a laser frequency of 35 Hz (200 shots). External calibration was performed using Peptide Calibration Standard II (Bruker Daltonics). Negative controls included omission of (i) RBD incubation, (ii) VHH_*E12*_ or C7C_18_ spotting, or (iii) both (membrane only). Peptide masses obtained from mass spectrometry were matched to theoretical masses derived from *in silico* tryptic digestion of the RBD sequence using the PeptideMass tool^2^. The sequence used for *in silico* analysis corresponded to the recombinant RBD construct (see Supplementary information 1). The same sequence was used to generate a 3D structure of recombinant RBD using the AlphaFold server (Abramson et al., 2024), which was visualized using Protein Imager (Tomasello et al., 2020).

## 3 Results

### 3.1 VHH-phage library and selection of RBD-binding VHH

**FIGURE 1:**
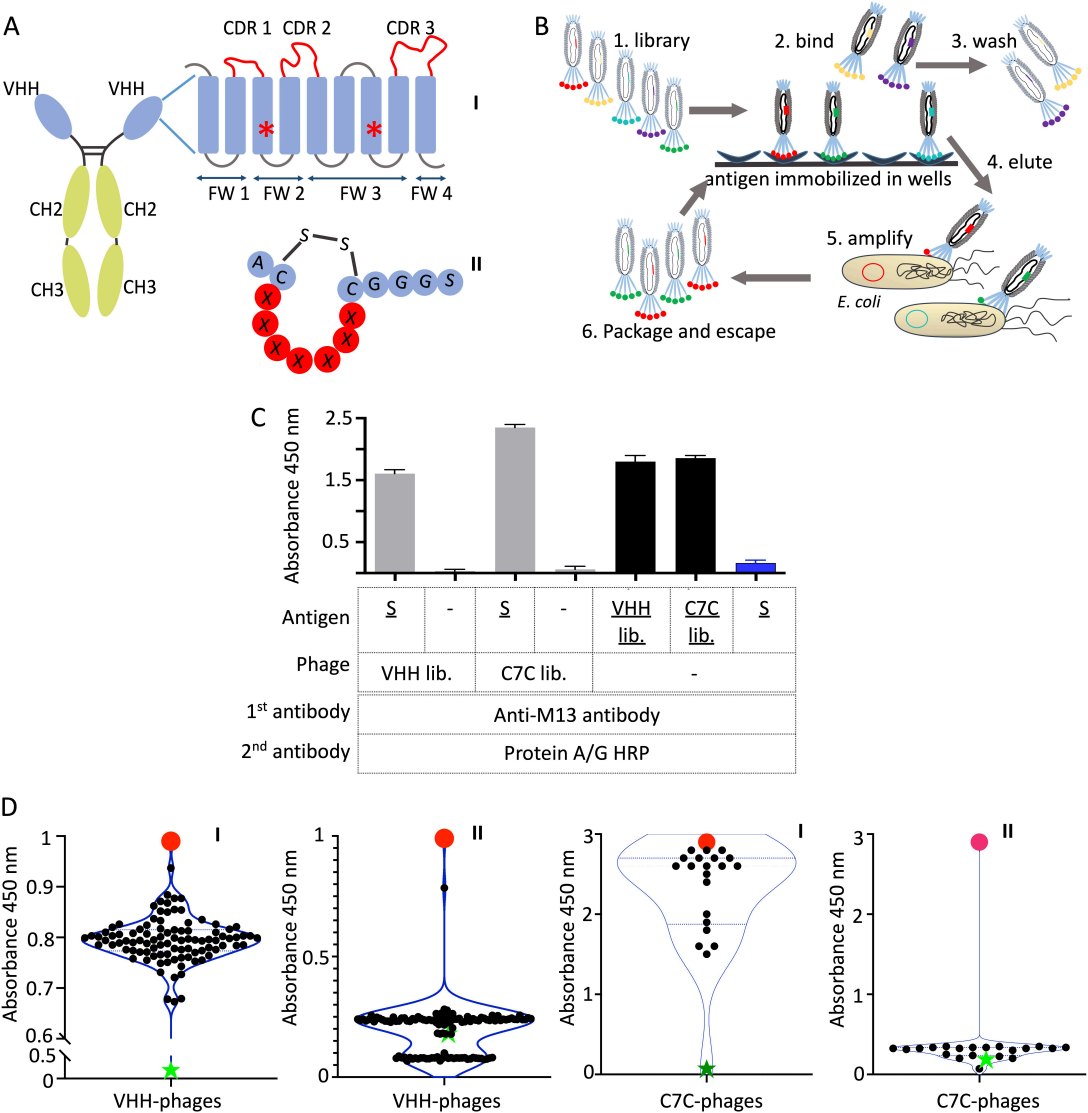
Phage display: selection of VHH-phages and C7C phages having affinity to Spike protein. **(A)** Illustration of the VHH (I.) and C7C (II.) molecules. Amplified VHH fragments in this study had 4 framework regions (FR1-4) and 3 complementarity-determining regions (CDR1-3). The C7C in combinatorial phage display consists of alanine-cysteine-7 random amino acids-cysteine. Cysteine in both VHH and C7C is essential for the formation of disulfide bond and proper folding. Cysteine residues present in frameworks are depicted with red asterisks. **(B)** Illustration of the panning–1. Phage library presenting either VHH or C7C on pIII protein. 2 and 3. Incubation of the phage library with antigen followed by rigorous washing. 4. Elution of bound phage. 5. Infection of *E. coli* with eluted phages for amplification, 6. Escape of the phages from *E. coli*, which is used in the next round of panning. **(C)** ELISA for assessment of binding of phages eluted from the last round of panning. S – spike protein, VHH lib. - VHH-phages eluted from the last round of panning. C7C lib. - C7C-phages eluted from the last round of panning. Underlined molecules were immobilized in the ELISA wells. **(D)** I. The binding affinity of individual VHH-phage or C7C-phage clones to Spike protein confirmed by ELISA. II. Spike protein was omitted from ELISA. Positive control (red dot)–phages coated on the well and detected by anti-M13 antibody; negative control (green dot)–phages were omitted from assay.

The recombinant spike protein, spanning the amino acids T^19^ to O^1208^, used to immunize *llama galma* lymphocytes *in vitro*, had a single band at ∼160 kDa in SDS-PAGE and showed a peak of the same size in MALDI-TOF MS (Supplementary information 1). The VHH amplified from immunized lymphocytes had approximately 500 bp (Supplementary information 2). The primers used to amplify VHH encompass all three CDR regions and four framework regions illustrated in Figure 1A. The amplified VHH sequences ligated into pJB12 phagemid and electroporated into *E. coli* generated approximately 1.3 × 10^11^ transformants (defined as the VHH-*E. coli* library). When only the digested phagemid (negative control) was electroporated, no transformants grew on the antibiotic-supplemented LB plate, suggesting that the VHH sequence is present in the majority of transformants in the VHH-*E. coli* library. The sequence analysis of the randomly picked transformants affirmed the presence of CDR and framework regions, and the distance matrix plot generated by comparing the amino acid sequences of VHH showed high sequence diversity (Supplementary information 2).

Superinfection of the *E. coli* library with helperphage resulted in packaging of phages that carry VHH on their minor coat protein pIII (as depicted in Supplementary information 2) and subsequently escape from *E. coli*. After NaCl-PEG precipitation of the escaped phages, approximately 3.9 × 10^14^ phages/mL were obtained, which was designated as the VHH-phage library. The phage library was used in 3 rounds of panning against recombinant RBD produced in this study (∼40 kDa in SDS-PAGE and MALDI-TOF MS, Supplementary information 1), which encompasses residues R^319^ to C^590^. The rationale for using RBD in panning was to choose RBD-specific VHH, which could potentially block binding of spike protein to the cell receptor because the interaction is predominantly mediated by the receptor binding domain. The use of full-length spike protein in panning may result in the generation of several VHH clones with affinity for spike protein domains other than the RBD, which may not impede virus attachment to cells.

After three rounds of the panning (as illustrated in Figure 1B), phages eluted from the last panning readily bind to the spike protein immobilized in the wells (Absorbance_*A450*_ 1.6 in phage ELISA, Figure 1C). Although panning was performed using the RBD, a full-length spike protein was used for coating in ELISA to ensure that the selected clones interact with the RBD present on the spike protein. In the absence of spike protein (wells blocked with bovine serum albumin only), no non-specific binding to plasticware or blocking agent was observed (A_450_ 0.08, Figure 1C).

### 3.2 C7C-phage library and selection of peptides

The commercially available Ph.D.-C7C phage display peptide library used here has seven randomized amino acids structurally constrained by a disulfide bond between cysteine residues present at both ends (forming a loop-like structure, Figure 1A). The diversity of the C7C-phage library is 1 × 10^9^, as declared by the manufacturer. After three rounds of panning performed as described above, enriched phages demonstrated the ability to bind spike protein (all phages A_450_ > 2.3 in phage ELISA, Figure 1C) and minimal binding to plasticware with A_450_ only 0.18.

### 3.3 Individual phage clones and their binding to spike protein

**FIGURE 2:**
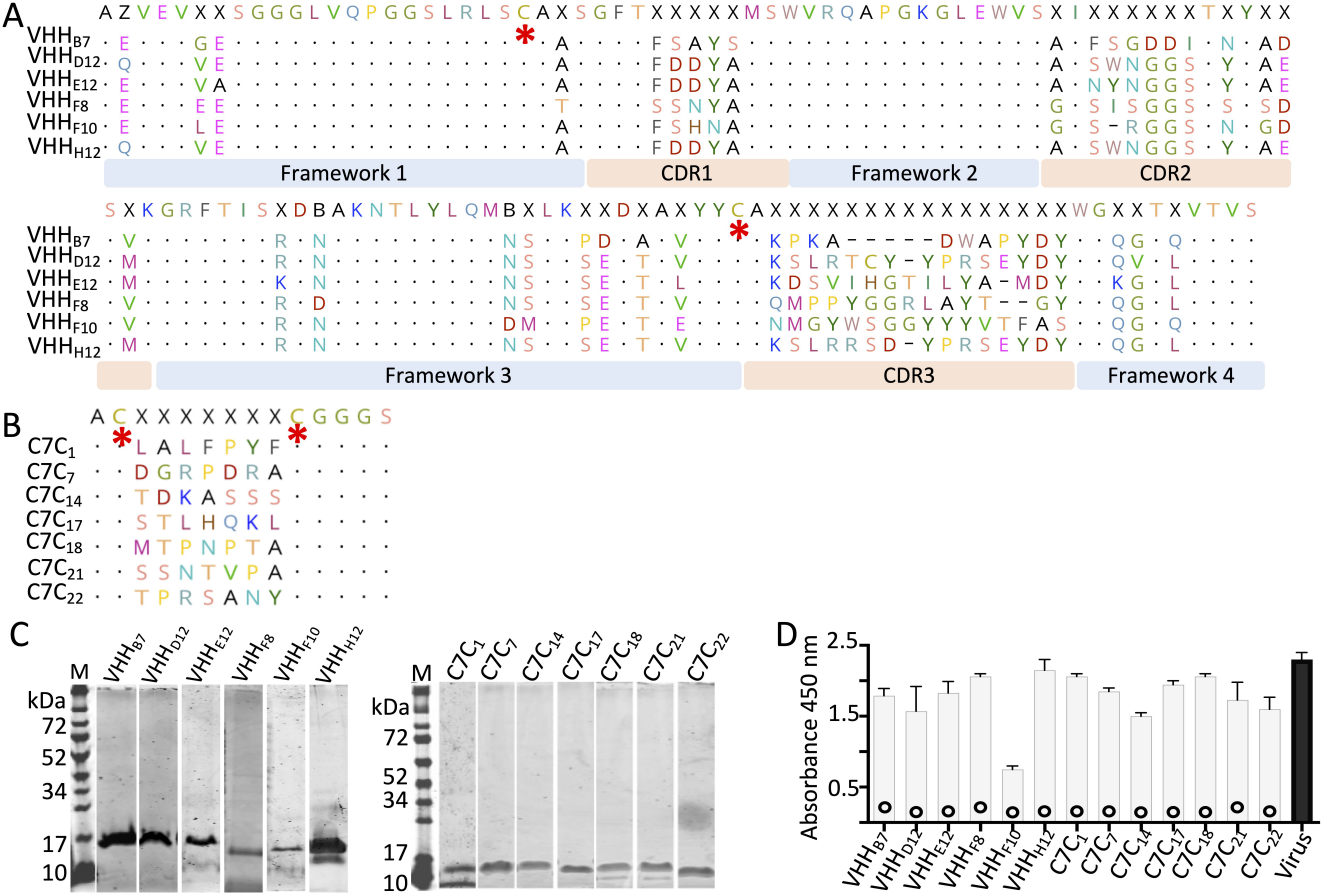
Phage clones, their soluble forms, and binding to spike protein. **(A)** Amino acid sequences of the representative VHH-phage clones from each cluster. Clusters were made based on sequence homology of randomly picked clones. Positions of frameworks and CDRs are shown. **(B)** Amino acid sequences of the representative C7C-phage clones from each cluster. Clusters were made based on sequence homology. In both **(A,B)** red asterisks indicate cysteine residue necessary for the formation of disulfide bond. **(C)** Purified VHH and C7C clones resolved on SDS-PAGE. Proteins were stained with Coomassie staining. **(D)** ELISA performed to assess binding of purified VHH and C7C clones with virions. The virions were fixed in the wells, incubated with 1 μg of each clone, and detected with nickel HRP conjugate. The absorbance values presented for VHH and C7C are. After subtraction of values obtained from background control (no virion). Rounds in each bar present absorbance in negative controls (VHH or C7C excluded from assay). Virus – indicate an input control (wells coated with virions were incubated with an anti-SARS-CoV-2 HRP antibody). Input control for VHH and C7C was performed by coating 1 μg of each molecule in the wells and detecting it with a nickel-HRP probe and Ultra TMB (absorbances for all input controls were >2.5; values are not depicted in graph).

Individual VHH-phages (*n* = 96) and C7C-phage (*n* = 20) clones demonstrated binding ability to spike protein in phage ELISA (Figure 1D, all clones A_450_ > 0.5). Meanwhile, the absorbance in positive and negative controls was A_450_ > 2.0 and <0.15, respectively (Figure 1D). Clones showing absorbance A_450_ > 0.8 were subjected to DNA sequencing and clustering based on amino acid sequence homology. VHH-phages formed six clusters, whereas C7C-phages were grouped into seven clusters (Figures 2A, B). A representative from each group was then produced in soluble form.

### 3.4 Soluble form of the VHH and C7C maintain the ability to bind spike protein

The purified soluble VHH (N-terminal 6x His) and C7C (N-terminal 6x His) had molecular sizes of between 14–17 kDa and 7.0–7.3 kDa, respectively (Figure 2C). All VHH and C7C candidates, except the VHH_*F10*_, showed the ability to bind virus in ELISA (A_450_ > 1.0, Figure 2D). None of the candidates showed non-specific binding when the virus was excluded from the assay. It is important to note that the VHH contains cysteine residues in framework 1 and 3 regions (Figure 2A), which are required for disulfide bond formation and thus proper folding. Similarly, the function of C7C relies solely on a loop structure formed by two cysteine residues flanking at both ends (Figure 2B). *E. coli* strains routinely used for protein production are not useful, as they possess a reducing cytoplasmic environment and lack post-translational modification. Thus, the *E. coli* strain Shuffle express, which possesses an oxidizing cytoplasmic environment that promotes disulfide bond formation (Lobstein et al., 2016), was used for VHH and C7C expression.

### 3.5 Soluble VHH and C7C restricted entry of pseudovirus in the cells

**FIGURE 3:**
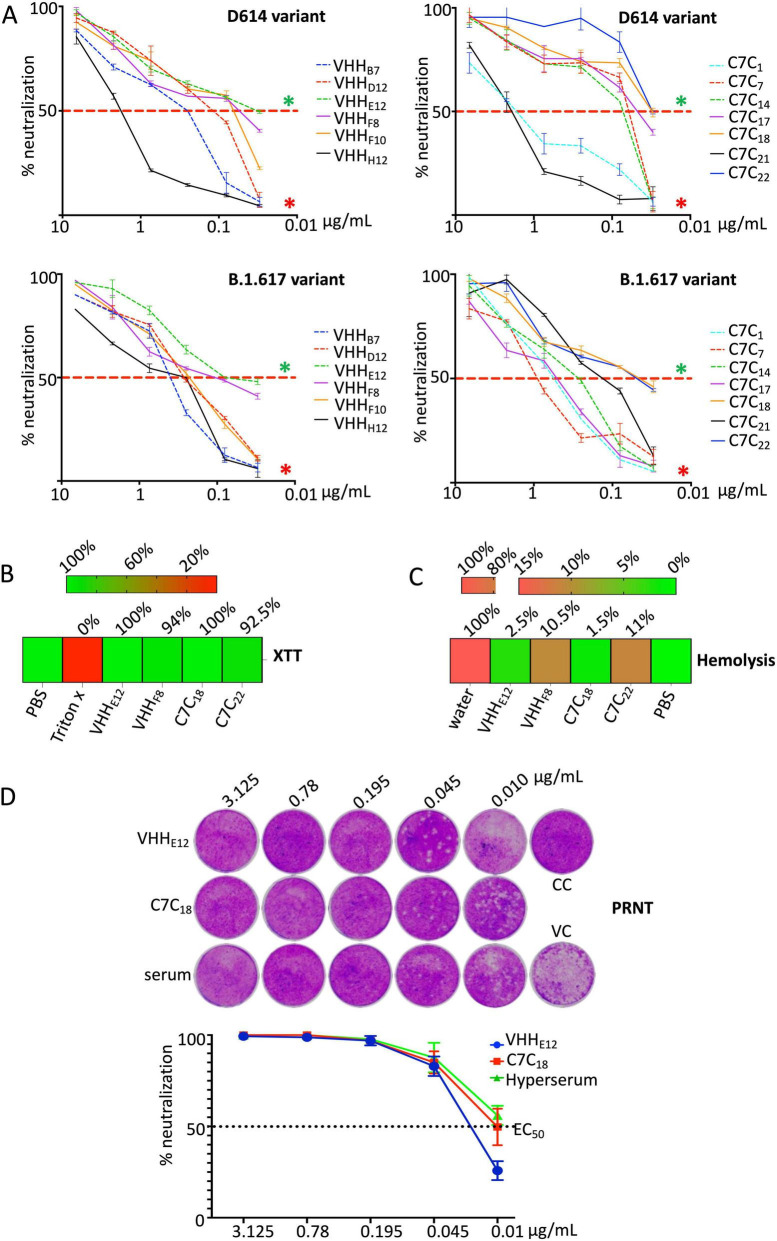
Neutralization of pseudovirus with VHH and C7C clones, their effect on cell metabolism and red blood cells, and PRNT of selected clones. **(A)** Neutralization of SARS-CoV-2 pseudovirus variants (D614 and B.1.617) by VHH and C7C clones. Hyperimmune serum (positive control, green asterisk) or PBS (virus control, red asterisk) used in the same assay indicates % neutralization. Serum was diluted similarly to VHH or C7C clones. The red line indicates EC_50_. **(B)** Effect of the VHH and C7C clones on cell metabolism assessed by XTT. PBS served as a control, which has no effect on cell metabolism, while Triton X served as a toxic agent. The scale bar indicates the % metabolism calculated based on non-treated cells. **(C)** Effect of the VHH and C7C clones on red blood cells (hemolysis). Water served as a control that causes hemolysis, while PBS served as a control that is tolerated by red blood cells. The scale bar indicates % hemolysis. **(D)** SARS-CoV-2 neutralization by VHH_*E12*_ and C7C_18_. Upper panel–representative images show stained plaques in the infected cells. Lower panel–a graph showing an average neutralization activity (%) of the tested compounds in each dilution (data normalized to virus control, i.e., no test compound added to virus before infection of the cells). The dotted black line represents 50% neutralization activity (PRNT_50_, or EC_50_), positive control–hyperimmune serum. Raw data. Absorbance values (XTT–panel B and hemolysis assays–panel C) and number of plaques (PRNT–panel D) used for normalization are provided in Supplementary data file 1.

The ability to block cell entry of pseudovirus was assessed by the pseudovirus neutralization test using two variants (D614 and B.1.617). Among all VHHs, VHH_*E12*_ demonstrated the best neutralization of D614, inhibiting VLP entrance at 0.04 μg/mL > 50%. VHH_*F8*_ was the second most effective VHH, inhibiting VLP entrance by more than 50% at 0.06 μg/mL. Although both VHH candidates were also the best inhibitors of B.1.617, more than 50% inhibition was achieved at higher concentrations (VHH_*E12*_ 0.09 μg/mL and VHH_*F8*_ 0.11 μg/mL, Figure 3A). In the case of cyclic peptides, C7C_18_ and C7C_22_ neutralized D614 pseudovirus entry by >50% at 0.03 μg/mL, while against B.1.617 the required concentrations were 0.04 and 0.05 μg/mL, respectively (Figure 3A).

### 3.6 Soluble VHH_*E12*_ and C7C_18_ are not toxic to cells and are hemocompatible

All four candidates (VHH_*E12*_, VHH_*F8*_, C7C_18_, and C7C_22_) with the best pseudovirus neutralization activity were tested for cell toxicity and hemocompatibility before being evaluated for the plaque reduction neutralization test. Cell metabolic activity was not significantly affected by any of the tested candidates (*p* > 0.01, two-tailed paired Student’s *t*-test) assessed with XTT. However, cells treated with VHH_*F8*_ (94% metabolic activity) and C7C_22_ (92.5% metabolic activity) showed a non-significant reduction in activity (Figure 3B).

In the hemolysis assay, incubation of VHH_*E12*_ and C7C_18_ with ovine red blood cells did not cause notable erythrolysis (<2.5% hemolysis compared to PBS, *p* > 0.01, two-tailed paired Student’s *t*-test); however, VHH_*F8*_ and C7C_22_ caused >10% hemolysis (Figure 3C), indicating that both molecules do not meet safety standards. Therapeutic agents are considered safe only when they do not reduce cell metabolic activity by more than 30% and do not cause hemolysis of more than 10%.

### 3.7 VHH_*E12*_ and C7C_18_ neutralizes the SARS-CoV-2 virus

VHH_*E12*_ and C7C_18_ were further tested for their ability to neutralize virus in a dose-dependent manner in a plaque reduction neutralization test using the VERO E6 cell line. 3.125 μg/mL of VHH_*E12*_ and C7C_18_ reduced the average number of plaques, reflecting 99% and 100% neutralizing activity, respectively. Although neutralization decreased with the increased dilution of both molecules, the highest dilution 0.01 μg/mL of VHH_*E12*_ and C7C_18_ exhibited the virus-neutralizing activities at 27.3% and 49.1%, respectively (Figure 3D). The hyperimmune serum, diluted in a similar manner to the test compounds, exhibited 55.5% neutralization in the last dilution (Figure 3D).

### 3.8 Fusion of VHH_*E12*_ and C7C_18_ with Angiopep-2 increases crossing across the BBB *in vitro*

**FIGURE 4:**
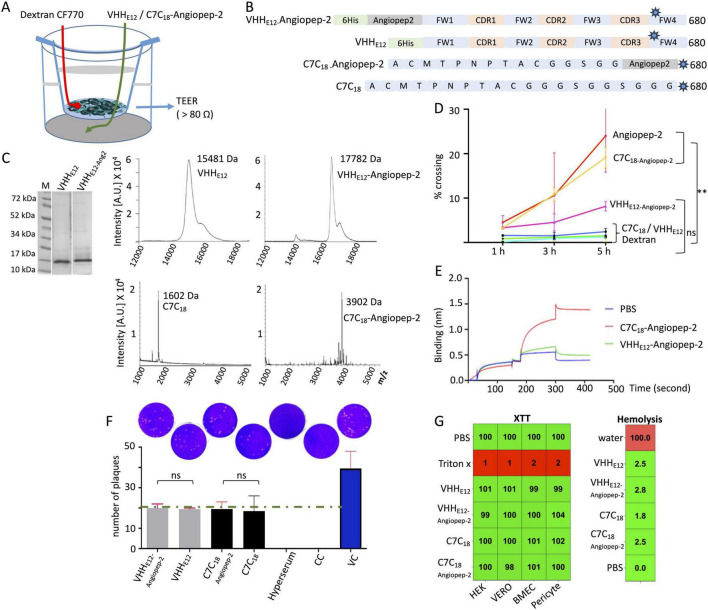
Variable Heavy domain of Heavy chain antibody (VHH) and C7C fused with Angiopep-2. **(A)** Illustration of the blood-brain barrier cultured *in vitro* in a cell insert, which is composed of upper (luminal) and lower (abluminal) chambers separated by a membrane with pores (1–3 micrometers) on which brain microvascular endothelial cells are cultured till the trans-endothelial electrical resistance (TEER) is achieved >80 Ω. The impermeability barrier to dextranCF770 also serves to check barrier integrity. Test compounds (VHH and C7C) can be added in the luminal chamber, and the % crossing of compounds to the lower chamber (BBB crossing) is measured based on fluorescence intensity. **(B)** Illustration of the VHHE12 and C7C18 fused with or without Angiopep-2. All molecules were tagged with infrared dye IRD680 (indicated by a blue asterisk). In the case of VHH, the His-tag and Angiopep-2 were fused at the N-terminus to keep the CDR3 (antigen-binding region) free. In the case of C7C, the Angiopep-2 was at the C-terminus, as the antigen-binding region is at the N-terminus. **(C)** VHH_*E12*_ and VHH_*E12*_ fused with Angiopep-2 resolved on SDS-PAGE (gel on the left). On the right-hand side in panel **(C)** the MALDI-TOF MS spectra of VHH_*E12*_ and C7C_18_ (with and without Angiopep-2 fusion) are presented. Molecular weights are depicted in Daltons (Da). For C7C peptides SDS-PAGE was not performed due to their small size. **(D)** Time-dependent crossing of VHH_*E12*_ and C7C_18_ (fused with and without Angiopep-2) through the BBB model. Positive control–Angiopep-2 Cy5.5; negative control–Dextran CF770. A significant difference in the crossing was observed in the case of C7C_18_ fused with Angiopep-2 (**p = 0.007) when compared to C7C_18_, while in the case of VHH_*E12*_ fused with Angiopep-2 it was non-significant (ns, *p* > 0.05, paired *t*-test – one tail, when compared to VHH_*E12*_). **(E)** Biolayer interferometry used to assess the binding affinity (KD) of angiopep-2 fused C7C_18_ and VHH_*E12*_ to recombinant RBD. KD C7C_18_ 1.009 × 10^– 6^, KD VHH_*E12*_ – 4.156 × 10^– 5^ M. **(F)** Neutralization of the SARS-CoV-2 virus by Angiopep-2 conjugated molecules. Both VHH_*E12*_ and C7C_18_ conjugated with Angiopep-2 did not show significant reduction in their ability to neutralize virus (ns, *p* > 0.05, paired *t*-test–one tail), when compared to their non-conjugated counterparts. CC – cell control (no virus added), VC – virus control (no preincubation of virus with any test compound before infection of the cells). **(G)** Effect of the VHH_*E12*_ and C7C_18_ (with and without Angiopep-2) on cell metabolism assessed by XTT. PBS served as a control, which has no effect on cell metabolism, while Triton X served as a toxic agent. Number in each cell presents % viability (metabolic activity). XTT was performed on 4 cell types viz. HEK293/17, VERO E6, brain microvascular endothelial cells (BMEC-5i), and pericytes (human brain vascular pericytes). Hemolysis–the effect of the VHH_*E12*_ and C7C_18_ (with and without Angiopep-2) on red blood cells. Water served as a control, which causes hemolysis, while PBS served as a control that is tolerated by red blood cells. The number in each cell presents % hemolysis. Raw data. Absorbance values (XTT and hemolysis assays) are provided in Supplementary data file 1.

We first tested the ability of VHH_*E12*_ and C7C_18_ to cross the BBB *in vitro*. The test was conducted using *in vitro* BBB models (grown on 24-well inserts, Figure 4A) that had the barrier characteristics, specifically transendothelial electrical resistance (TEER) between 100 and 120 Ω/cm^2^ and crossing of dextran-CF770 from luminal to abluminal chambers < 5% after 5 h. Dextran crossing of less than 5% signifies that the barrier is well formed and devoid of fenestrae. We observed that only 0.9% of the VHH_*E12*_ was able to cross the barrier, while in the case of C7C_18_ it was 1.6% after 1 h of incubation. The crossing was increased only to 1.1% for VHH_*E12*_ and 1.8% for C7C_18_ after 5 h (Figure 4D).

To increase the translocation of VHH and C7C, we fused them with Angiopep-2, the well-known CNS homing peptide (Figures 4B,C). In the case of VHH_*E12*_-Angiopep-2 and C7C_18_-Angiopep-2, 3.3% and 3.2% of the crossing was recorded after 1 h, which increased to 8.1% (an 5.4-fold increase compared to VHH_*E12*_) and 19.1% (a 11.2-fold increase compared to C7C_18_) after 5 h, respectively (Figure 4D). In the control reaction, the Angiopep-2 was crossed at a rate of 4.5% after 1 h and 24% after 5 h. The findings demonstrate that conjugation of C7C with Angiopep-2 significantly increased its transport across the BBB; however, in the case of VHH, although the translocation was increased by 5.4-fold, the difference was not statistically significant.

The fused molecules were further assessed for their binding affinity to RBD. The biolayer interferometry revealed that the dissociation constant between Angiopep-2 fused C7C_18_ and RBD was 1.009 × 10^–6^ M and between VHH_*E12*_ and RBD it was 4.156 × 10^–5^ M (Figure 4E).

### 3.9 Fusion of VHH_*E12*_ and C7C_18_ with Angiopep-2 did not cause a reduction in the virus neutralization capacity and did not alter the cytotoxicity or hemocompatibility

Further analysis of VHH_*E12*_-Angiopep-2 and C7C_18_-Angiopep-2 revealed that the fusion constructs maintained their virus neutralization capacity when tested against SARS-CoV-2 (lineage BA.5) (Figure 4F). Both Angiopep-2 conjugated molecules maintained their non-hemolytic property and were not toxic to the cells (Figure 4G).

### 3.10 Plausible binding sites for VHHE12 and C7C18 on RBD

**FIGURE 5:**
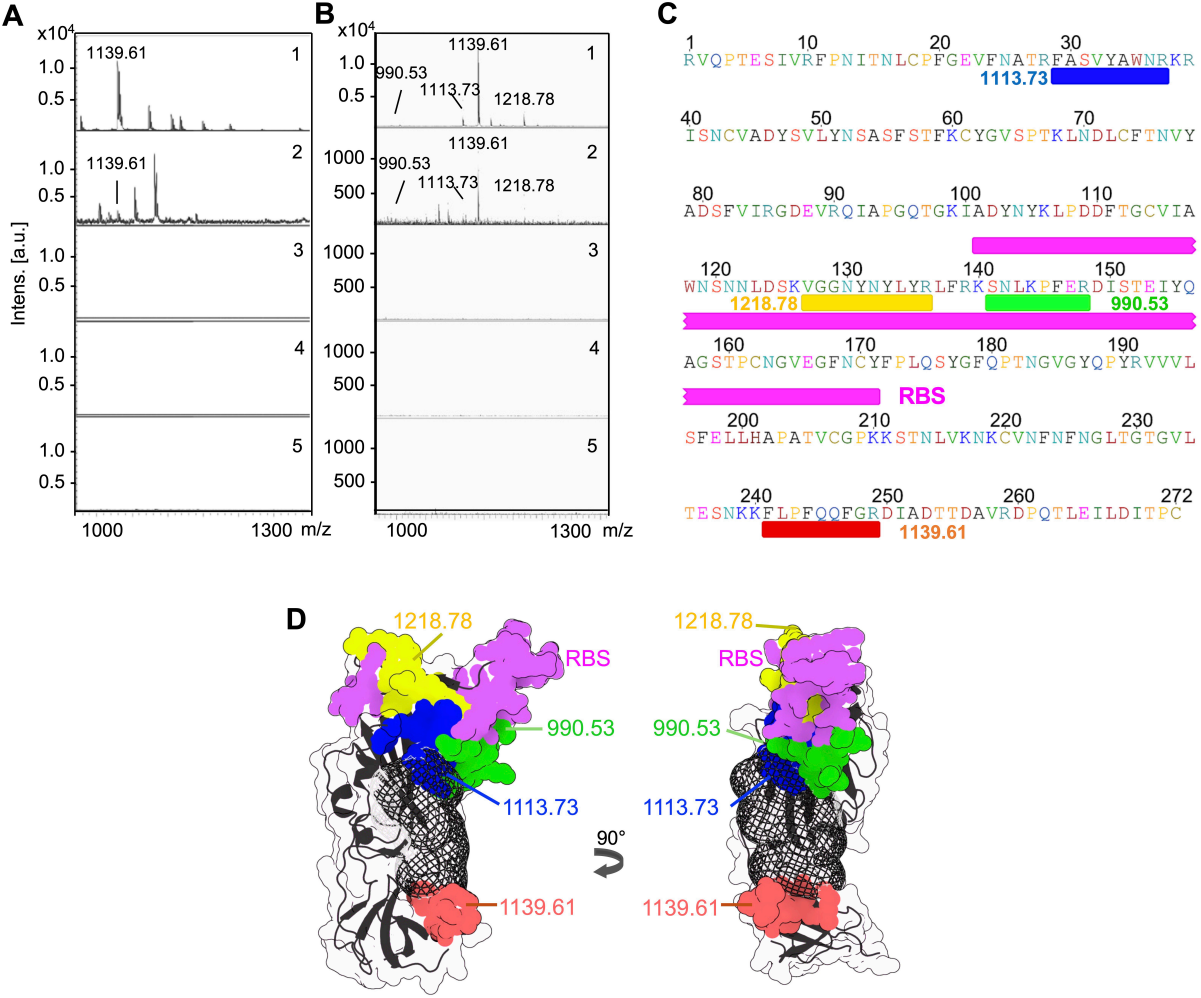
Identification of plausible VHH and C7C epitopes on RBD. **(A)** Identification of peptides of RBD plausibly corresponding to the VHH epitope (VHH was immobilized on the membrane). Identification was performed with limited tryptic digestion of the VHH-RBD complex. Spectrum 1: MALDI-TOF spectrum of in-solution limited tryptic digestion (1 h) of RBD. 2: VHH was immobilized on PVDF membrane, allowed to interact with RBD, the complex was trypsinized, and peptides of RBD bound to VHH were retrieved (eluted) and analyzed on MALDI-TOF-MS. 3: Negative control – RBD was omitted from the assay shown in 2. 4: Negative control – VHH was omitted from the assay shown in 2. 5: Negative control - VHH and RBD were omitted from the assay. **(B)** Identification of peptides of RBD plausibly corresponding to the C7C epitope (C7C was immobilized on the membrane). 1: MALDI-TOF spectrum of in-solution limited tryptic digestion (1 h) of RBD. 2: C7C peptide was immobilized on PVDF membrane, allowed to interact with RBD, the complex was trypsinized, and peptides of RBD bound to C7C were retrieved and analyzed on MALDI-TOF-MS. 3: Negative control – RBD was omitted from the assay shown in 2. 4: Negative control – C7C was omitted from the assay shown in 2. 5: Negative control – C7C and RBD were omitted from assay. **(C)** Amino acid sequence of RBD (Supplementary information 1) with highlighted receptor binding site (magenta) and peptides identified as interacting sites. **(D)** Crystal structure of RBD (Alphafold) in which the plausible binding site of VHH is highlighted (1139.61, red) along with the “silent face” (mesh) and RBS (faint magenta). Crystal structure also shows plausible binding sites of C7C (yellow, blue, and green).

Plausible binding sites of VHH_*E12*_ and C7C_18_ on RBD were determined using limited proteolysis of VHH-RBD and C7C-RBD complexes on PVDF membrane (Figures 5A,B). With this approach, 4 peptides were obtained from the VHH-RBD complex, among which the 1139.61 Da was showing an exact match with the peptide obtained after limited proteolysis of the RBD only (Figure 5A, spectra 1 and 2). The sequence of this peptide is FLPFQQFGR as observed in the theoretical masses derived from *in silico* tryptic digestion of the RBD sequence (Figure 5B). The position of this peptide in the amino acid sequence of RBD indicated that it lies downstream of receptor binding site, and mapping of this peptide on the 3D structure revealed its proximity to so-called “silent face” of RBD (Figure 5D), which is less prone to mutations than dominant RBD sites, acting as a crucial target for broadly neutralizing antibodies (Bajpai et al., 2022; Liu and Wilson, 2022).

Limited proteolysis of the C7C-RBD complex revealed four peptides (Figure 5B, spectrum 2), among which showed an exact match with the peptides derived from the limited proteolysis of the RBD alone, and were present in the list of theoretical masses of the *in silico* tryptic digest of RBD. Peptides 990.53 Da (SNLKPFER) and 1278 (VGGNYNYLYR) are colocalized on the 3D structure, adjacent to the “silent face” of RBD, with the other two peptides 1113.73 Da (FASVYAWNR) and 1139.61 Da (FLPFQQFGR) obtained from the C7C-RBD complex (Figures 5C,D). The 1139.61 Da peptide was also obtained from VHH-RBD complex. The peptides identified in the mass spectrometry were derived specifically from the VHH-RBD and C7C-RBS complexes, as no nonspecific peptides were leached out from the PVDF membrane when either VHH or C7C or RBD was omitted from the assay (Figures 5A,B, spectra 3, 4, and 5). Other eluted peptides did not correspond to any *in silico*-generated peptides and remained unidentified.

Mapping of a plausible VHH binding site on spike RBD revealed that VHH might target the “silent face” of RBD, also known as the lateral site. This antigenic site remains hidden in RBD-down conformation and is becoming accessible only upon transition to RBD-up state, which is necessary for ACE2-binding (Valério et al., 2022; Song et al., 2025). Generally, antibodies targeting the silent face of RBD are considered more broadly neutralizing and resistant to antigenic shift resulting from the high mutational rate in the RBD (Bajpai et al., 2022; Liu and Wilson, 2022). Our results suggest that VHH_*E12*_ binds to the silent face of RBD, which explains its ability to neutralize multiple SARS-CoV-2 variants used in this study. Mapping of plausible C7C binding site on RBD using the same assay yielded four peptides, with three colocalizing on the RBS of RBD. Direct blocking of RBS by C7C peptides would halt Spike protein binding to ACE-2, resulting in efficient neutralization.

## 4 Discussion

Over the course of the COVID-19 pandemic, several variants of SARS-CoV-2 have emerged, causing increased transmission throughout the world. Several variants are designated as “*variants being monitored*,” while Delta and Omicron are the “*variant of concerns*” – VOC (Centers for Disease Control and Prevention [CDC], 2021). Several mutations have been identified in the spike protein of VOC, particularly in the Delta variant (B.1.617.2), which may have altered the organ tropism and raised the likelihood of neurological sequelae following Alpha, Delta, or Omicron infection (Taquet et al., 2022; Proust et al., 2023). Since 2021, the incidence of neurological complications and the long-COVID-19 is evident. In one study, nearly 70% of long-COVID was recorded among hospitalized patients (Davis et al., 2023). Another case study from a cohort of 321 patients revealed that the prevalence of long-COVID was as high as 61% (Maestre-Muniz et al., 2021). Some variants are predicted to enter the CNS via the olfactory and other cranial nerves, as well as through infection of the brain microvascular endothelial cells (Pattanaik et al., 2023). Infection of the endothelial cells of brain microvasculature is a serious concern, as it can compromise the integrity of the BBB. Studies have shown that SARS-CoV-2 infects astrocytes (Crunfli et al., 2022), and is found in the cerebrospinal fluid and brain tissue of COVID-19 patients (Ellul et al., 2020; Paniz-Mondolfi et al., 2020; Stein et al., 2022).

Recent studies have attempted to develop anti-SARS-CoV-2 therapies that can cross the BBB and exert virus neutralizing effects. Molnupiravir and its metabolite β-D-N4-hydroxycytidine can penetrate the blood-brain barrier in 20 min when taken with an efflux inhibitor (Chang et al., 2023). Co-administration of another anti-COVID-19 medication, nirmatrelvir with *Scutellaria baicalensis* extract, was demonstrated to efficiently traverse the BBB (Lee et al., 2024). However, antiviral nucleoside treatment, such as molnupiravir, may result in thrombocytopenia and host DNA damage (Zhou S. et al., 2021). To this background, it is necessary to fortify therapeutic measures against CNS infection caused by SARS-CoV-2. The small size of the VHH and cyclic peptides, when compared to conventional IgG, allows for easy conversion into the molecular system for enhanced biodistribution in the brain parenchyma. Other benefits of smaller antigen-binding motifs include the potential for chemical synthesis and easy structural optimization. For instance, PepC7, the cyclic peptide consisting of 7 amino acids, thanks to its small size, showed approximately 41-fold higher BBB transcytosis compared with the full-sized phage particle (Zhou X. et al., 2021). Recently we reported facile modification of the C7C peptide to enhance BBB crossing by fusing the homing peptide (Mochnacova et al., 2025), and decoration of the VHH on the dendrimer-Angiopep-2 against CNS infection caused by tick-borne encephalitis virus (Jozefiakova et al., 2025). Several other homing peptides and receptor-mediated transport systems, such as TGN, TfRL, ApoE, and PepH3, have been explored for effective transportation of peptides, VHH, and nanoparticles (Qian et al., 2025). We adopted Angiopep-2 in this study because of the absence of cysteine residues in its sequence, which may otherwise interfere with the disulfide bond formed between two cysteine residues present in the VHH or C7C. In the pilot experiment of the study, the fusion of TfRL (Transferrin Receptor Ligand, sequence: N-THRPPMWSPVWP) at the N terminus of VHH was also attempted; however, it resulted in excessive precipitation of the TfRL-VHH, resulting in the loss of nearly 95% of overexpressed protein (author’s unpublished data). Another rationale for using Angiopep-2 in this study was its non-toxicity, rigorously tested biodistribution attributes *in vivo*, and inclusion in drug delivery systems that are already being investigated in clinical trials (Kumthekar et al., 2020; Habib and Singh, 2022). Most importantly, thanks to its human origin (derived from human aprotinin), it typically displays very low immunogenicity, and it is generally considered safe (Habib and Singh, 2022). Angiopep-2 was successfully employed by us to transport the VHH-fused dendrimer nanosystem over the BBB (Jozefiakova et al., 2025); therefore, knowing its favorable biological properties, it was the primary choice to fuse VHHE12 and C7C18.

In this study, we adopted a strategy that relies on the inhibition of viral entry into the host cell by preventing the docking of spike protein on the ACE2 receptor. Use of the receptor-binding domain in the panning is preferred when the overall strategy of the development of the biomolecule is to block the attachment of viral protein to the cell receptor. In the recent studies, we and others have efficiently used the receptor binding domain of various viruses, such as tick-borne encephalitis virus, West Nile virus, and SARS-CoV-2, to develop VHH with neutralizing capacity at nanomolar to sub-nanomolar concentrations (Hanke et al., 2020; Huo et al., 2020; Jozefiakova et al., 2025; Petrouskova et al., 2025). In general, the RBD and spike proteins remain the primary antigen targets of SARS-CoV-2 for the production of nanobodies (Custodio et al., 2020; Dong J. et al., 2020; Esparza et al., 2020; Hanke et al., 2020; Schoof et al., 2020; Wu et al., 2020). Some nanobodies have nanomolar range EC_50_, such as clone Ty1 binding RBD with EC_50_ of 0.77 nM (Hanke et al., 2020), clone R3DC23 exhibiting EC_50_ of ∼0.8 nM (De Cae et al., 2025) and clone H11-H4 developed against RBD neutralizing virus at EC_50_ between 4 and 6 nM (Huo et al., 2020). The nanobody VHH_*E12*_, developed in this study, neutralized pseudovirus of D614 variant with >50% at 0.02 μg/mL corresponding to 1.18 nM (Figure 3A), which is in the sub-nanomolar range and comparable to other anti-SARS-CoV-2 nanobodies reported above. The same nanobody required higher concentration (0.06 μg/mL, 3.56 nM) to neutralize B.1.617 variant pseudovirus by more than 50%. At 0.045 μg/mL concentration, VHH_*E12*_ neutralized live Omicron (lineage BA.5) by more than 75% in PRNT. In the case of C7C_18_, the concentration required to neutralize pseudoviruses > 50% (EC_50_) was similar to that seen in VHH_*E12*_ (Figure 3A). The C7C_18_, even at a higher dilution of 0.01 μg/mL, neutralized Omicron by more than 50% in PRNT (Figure 3D). Please note that we were not able to perform PRNT against D614 and B.1.617, because of the unavailability of viral strains in our (BSL-3) biocontainment facility.

Both VHH_*E12*_-Angiopep-2 and C7C_18_-Angiopep-2 were tested for their toxicity, as therapeutic agents are considered safe only when they do not affect proliferation, do not reduce cell metabolic activity by more than 30% (ISO10993-5:2009(E), 2009), and do not cause hemolysis of more than 10% (Amin and Dannenfelser, 2006). Both tested molecules demonstrated all safety characteristics. None of these two molecules showed signs of cell toxicity even at 20 μg/mL and hemolysis at 10 μg/mL, which is a multiple-fold higher concentration tested in both assays than the concentration required to neutralize SARS-CoV-2 in PRNT.

Traditionally, antibody or nanobody development against pathogens entails immunization of animals with live or attenuated agents, which poses threats to the environment and exposes researchers to infectious hazards while also requiring significant time, labor, and animal biocontainment facilities. To address these challenges during a global emergency, we have developed a pipeline to induce the llama lymphocytes *in vitro* and use them as a source of mRNA to construct the VHH-phage library for downstream selection of VHH (Comor et al., 2017). The same *in vitro* immunization strategy was applied in the present study, followed by the phage display and rigorous selection of the best VHH candidates, resulting in the production of VHH_*E12*_ with the ability to neutralize the virus at sub-nanomolar concentrations. Combinatorial phage display, such as C7C-phages, which was used in this study to generate RBD-specific peptides, provides an additional option for rapid engineering of an effective neutralizing molecule against highly contagious pathogens.

Rapid engineering of biomolecules using phage display may have several drawbacks, such as toxicity or immunogenicity of developed molecules and cumbersome translation of phage display-derived peptides/antibodies into clinical trials, as the peptides displayed on the phages may not always maintain their activity in their synthetic form. In several experimental setups, phage display-derived peptides and antibodies may exhibit low binding affinity to the target, primarily due to biopanning bias, which occurs when multiple rounds of the selection start to favor clones with higher display efficiency or faster growth rates, rather than just the highest binding affinity. In the present study, binding affinity for Angiopep-2 fused C7C_18_ was measured in subnanomolar range (1.009 × 10^–6^ M), while, the affinity for VHH_*E12*_ was only 4.156 × 10^–5^ M (Figure 4E). The low binding affinity to the target could also be because phage display technology does not offer post-translational modifications, mainly glycosylation, which is necessary to make antibodies functional. The current study also lacks testing of developed anti-SARS-CoV-2 molecules in an *in vivo* setting, which is critical when it comes to the drug’s biodistribution in the CNS. *In vitro* models can provide preliminary data on the ability of the test molecule to traverse the BBB; nevertheless, one should be cautious when interpreting the data, because translation of the molecules can be affected by multiple factors and fidelity of models. Low/moderate fidelity models (2D static transwell with brain endothelial cells, used in the present study) can provide good data on the relative ability of translocation of tested molecules (e.g., peptides fused with Angiopep-2 vs. without fusion); however, these models lack fluid shear stress and co-culture systems (e.g., in high fidelity models –microfluidic 3D “on-a-chip”), which can replicate *in vivo* permeability. Thus, molecules developed in this study warrant further assessment in an *in vivo* model.

RNA viruses like SARS-CoV-2 have a high mutation rate, which contributes to their rapid evolution. As of spring 2026, several variants of SARS-CoV-2, including a new heavily mutated variant known as BA.3.2 (or nicknamed “Cicada”), are circulating globally. It should be noted that mutations in the RBD may impair the effectiveness of vaccines and therapeutics (including monoclonal antibodies) against infection. This warrants testing of therapeutic molecules already in clinical phase or in the development pipeline, against new variants. In the present study, we could not perform PRNT on various SARS-CoV-2 variants, such as Cicada, mainly due to the lack of new variants in our BSL-3 biocontainment facility.

## 5 Conclusion

In the current study, VHHs targeting the RBD of the spike protein of SARS-CoV-2 were developed using a well-validated experimental pipeline that included *in vitro* immunization of llama lymphocytes, VHH-*E. coli* and VHH-phage library construction, and panning for rigorous selection of VHH that plausibly block interaction between spike protein and cell. Combinatorial phage display was also used, reducing the time and work required for immunization and library construction. VHH_*E12*_ and C7C_18_ successfully neutralized SARS-CoV-2 at sub-nanomolar concentrations. Both were non-toxic and not hemolytic. Fusion of VHH and C7C with Angiopep-2 enhanced translocation of molecules across the BBB *in vitro*. Given the favorable features, we believe that both fusion molecules could be promising candidates for testing *in vivo* and in preclinical research.

## Data Availability

The datasets presented in this study can be found in online repositories. The names of the repository/repositories and accession number(s) can be found below: https://www.ncbi.nlm.nih.gov/genbank/, (VHH-CloneB7 PX942682, VHH-CloneD12 PX942683, VHH-CloneE12 PX942684, VHH-CloneF8 PX942685, VHH-CloneF10 PX942686, VHH-CloneH12 PX942687).

## Footnotes

1
www.graphpad.com

2
https://web.expasy.org/peptide_mass/
