# Supplementary material for: Anti-SARS-CoV-2 VHH and C7C peptide fused with Angiopep-2 efficiently traverse blood-brain barrier model and neutralizes virus
Source: Front Microbiol. 2026 Jun 18;17:1827887. doi: 10.3389/fmicb.2026.1827887 (PMC13323710; doi:10.3389/fmicb.2026.1827887)
Supplement: Supplementary file 1 [file Supplementary_file_1.pdf]

Anti-SARS-CoV-2 VHH and C7C peptide fused with Angiopep-2 efficiently traverse blood-brain barrier model and neutralizes virus

Mangesh Bhide[1,2,*], Katarina Bhide[1#], Evelina Kanova[1#], Jakub Viglasky[1], Diana Kosturikova[1], Amod Kulkarni[1,2]

[1]Laboratory of Biomedical Microbiology and Immunology, University of Veterinary Medicine and Pharmacy in Kosice, Slovakia.

[3]Institute of Neuroimmunology of Slovak Academy of Sciences, Bratislava, Slovakia.

# equal contribution

Table of Contents

Supplementary information 1. Production of recombinant spike protein and receptor-binding domain (RBD) ... 2
Supplementary information 2. VHH-phage library ... 6
Supplementary information 3. Panning—Isolation of C7C peptides ... 10
Supplementary information 4. Phage ELISA ... 11
Supplementary information 5. Isolation of individual VHH-phage and C7C-phage clones ... 12
Supplementary information 6. Phage ELISA for individual clones ... 13
Supplementary information 7. Isolation of phage ssDNA and sequencing. ... 14
Supplementary information 8. Production of soluble VHH and C7C ... 15
Supplementary information 9. Pseudovirus (virus like particle, VLP) titration and their pseudovirus neutralization assay ... 17
Supplementary information 10. Plaque reduction neutralization test (PRNT) ... 19
Supplementary information 11. Production of VHH and C7C fused with Angiopep-2 ... 20
Supplementary information 12. Virus neutralization test of the Angiopep-2 conjugated VHH and C7C ... 22
Supplementary information 13. Crossing of *in vitro* blood-brain barrier ... 23

Supplementary information 1. Production of recombinant spike protein and receptor-binding domain (RBD)

Cloning of the synthetic genes encoding spike protein and RBD, expression of proteins and purification. Synthetic genes encoding spike protein and RBD were cloned into pCMV-based mammalian expression vector and the plasmid DNA was amplified from transformed *E. coli* DH5α strain (ThermoFisher Scientific, Slovakia) grown overnight at 37 °C in LB (Luria-Bertani) medium (Sigma-Aldrich, Germany) supplemented with kanamycin (25 µg/mL; Duchefa Biochemie, Netherlands). Plasmid extraction and purification was performed using PureLink® HiPure Plasmid Filter Maxiprep Kit (ThermoFisher Scientific). Next, the purified plasmid (100 µg/mL) was electroporated into 2.1 x 10^8 cells/mL of human cell line Expi293 (ThermoFisher Scientific) resuspended into electroporation buffer using MaxCyte STX (MaxCyte, MD, USA). After 30 min of resting time, the electroporated Expi293 cells were diluted to obtain the final concentration of 3.1 x 10^6 cells/mL and incubated with shaking at 37 °C for 24 h. Thereafter, the culture was incubated for about 4 - 6 days at 32 °C thereby reducing the cell viability to 50% of the initial concentration. The reduced culture was centrifuged at 300 x *g* for 12 min and the resulting cell culture medium containing secreted recombinant protein was stored at -20 °C until further purification on ÄKTA purifier (GE-healthcare, Chicago, USA). In short, the cell culture medium was re-centrifuged at 20000 *x g* and the clarified supernatant was added with 0.5 M NaCl (Sigma-Aldrich, Germany) and filtered through 0.2 µm membrane filter. The filtrate was subjected to Nickel affinity chromatography on ÄKTA purifier using 5 mL Cytiva His-Trap™ affinity column (Thermo Fisher Scientific). After stringent washings with 4 column volume of 20 mM sodium phosphate buffer (pH 7.4), the His-tagged protein was eluted by 0.5 M imidazole in 20 mM sodium phosphate buffer (pH 7.4). Thereafter the eluted protein was further purified on Strep-Tactin® affinity purification system (5 mL Strep-tag®II column; IBA GmbH, Germany) using 10 mM desthiobiotin elution solution. Lastly, the purified proteins – recombinant spike (r-spike) and recombinant receptor binding domain (r-RBD) were subjected to HiTrap™ Desalting column (GE Healthcare) to perform buffer exchange into phosphate buffer saline (PBS, pH 7.4) and filter sterilized using 0.2 µm syringe filter.

The recombinant spike protein (S) contains residues from T^{19} to O^{1208} (GenBank: MN908947). Mutations K^{98}-P^{986}, V^{987}-P^{987} were introduced to achieve perfusion stability and furin cleavage site was substituted with $GSAS^{682–685}$). Recombinant spike also contained T4 fibritin trimerization motif, HRV3C protease cleavage site, 8X his-tag and a twin-Strep-tag at the C-terminus (**see below panel A**). The RBD spanned residues R^{319} to C^{590} of spike protein and it contained HRV3C protease cleavage site, 8X His-tag and twin-Strep-tag at C terminus (**see below panel B**).

Panel A

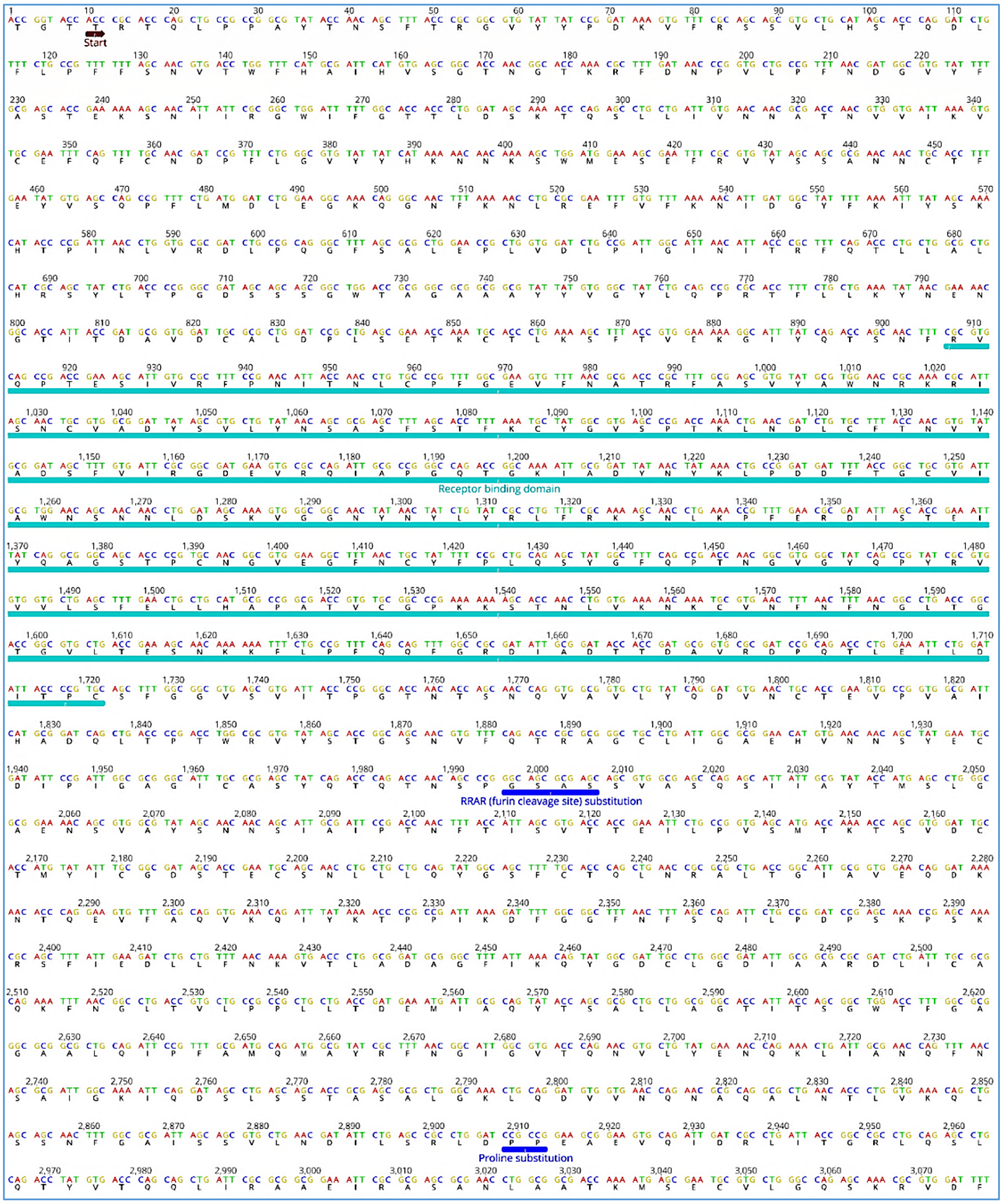
Start
Receptor binding domain
RRAR (furin cleavage site) substitution
Proline substitution

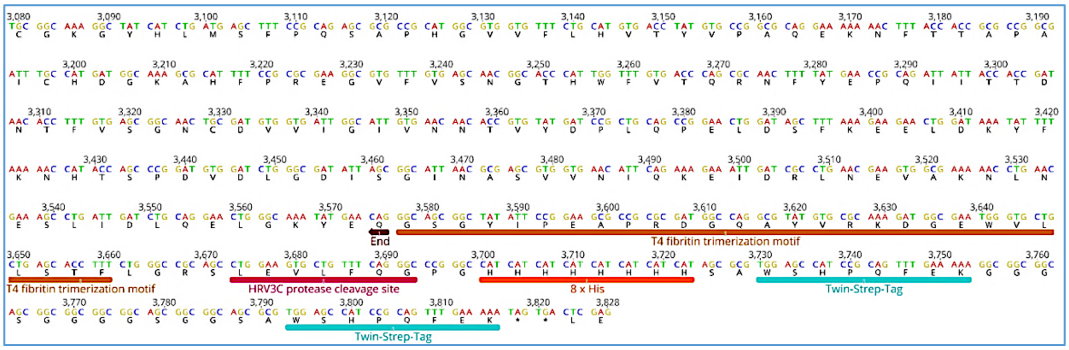

Panel B

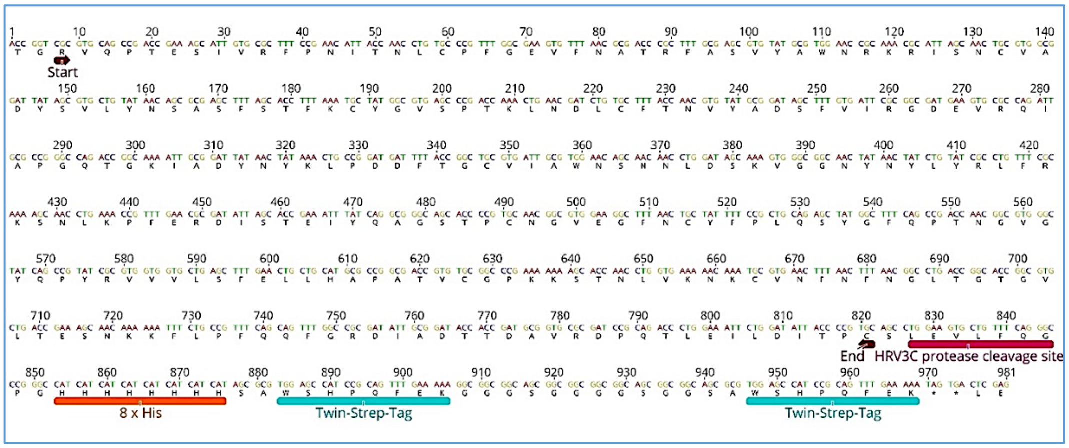

Sequence of spike protein and its receptor binding domain (RBD) used to produce recombinant proteins. Panel A. *Nucleotide and its corresponding amino acid sequence of spike protein (GenBank: MN908947) with marked annotations for – start and end of target sequence, receptor binding domain, furin cleavage site substitution, proline substitution (for perfusion stability), T4 trimerization motif, HRV3C protease cleavage site, 8X His (Histidine tag), and Twin step tag (streptavidin tag). Panel* ***B.*** *Nucleotide and its corresponding amino acid sequence of receptor binding domain (RBD) with marked annotations - start and end of target sequence, HRV3C protease cleavage site, 8X His (Histidine tag), and Twin strep tag (streptavidin tag).*

Quality control of the recombinant spike protein and RBD. The protein samples (15 µL each) were mixed with lithium dodecyl sulfate sample buffer (4X LDS sample buffer; Invitrogen, Slovakia) as per the manufacturer's instructions and incubated at 72 °C for 10 min. Electrophoresis was carried out at 30 mA in 1X running buffer (20X NuPAGE MOPS SDS running buffer, Invitrogen) until the dye reached the bottom of the gel (12% Bis-Tris polyacrylamide 12 well gel, Invitrogen). Proteins were stained with Coomassie staining (Bio-Rad Laboratories, USA). Results of LDS-PAGE are in shown below in **panel A**.

For MALDI-TOF MS, 0.8 µL of the purified proteins were mixed with 0.8 µL sDHB matrix (2,5-2,5-dihydroxybenzoic acid and 2-hydroxy-5-methoxybenzoic acid, Bruker Daltonics, USA) dissolved up to saturation in TA50 (50:50 [v/v] acetonitrile: 0.1% trifluoroacetic acid, TFA; Sigma-Aldrich). One microliter of the protein-matrix mix was spotted on the GroundSteel plate (Bruker Daltonics) and allowed to air dry. The acquisition was performed in flexControl v3.4 (Burker-Daltonics) in linear mode with 60 Hz laser intensity (200 shots) on MALDI-TOF MicroflexTM-LRF mass-spectrometer (Bruker Daltonics). Mass was analyzed in

flexAnalysis v3.4 software (Bruker Daltonics) by comparing it with calibrants (Protein calibration kit I, Bruker-Daltonics). Results of MALDI-TOF are below in **panels B and C**.

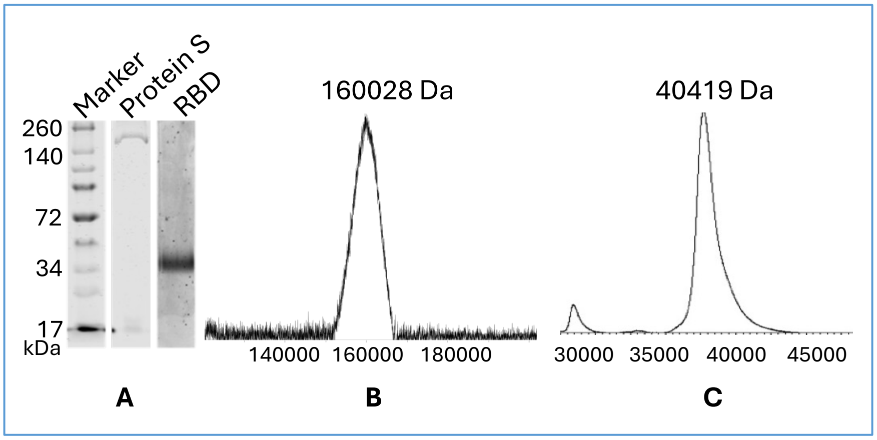

Production of the recombinant forms of spike protein and RBD. *Panel A. shows purified recombinant spike protein and RBD analyzed by LDS-PAGE. Panel B. Determination of the molecular mass of the spike protein by MALDI-TOF, showing a measured mass of ~160 kDa. Panel C. Determination of the molecular mass of the RBD by MALDI-TOF.*

Supplementary information 2. VHH-phage library

The alpaca lymphocytes were in vitro immunized with spike protein exactly as described in our previous publication (Comor et al., 2017). After immunization, the RNA was isolated using RNeasy mini kit (Qiagen), and cDNA was synthesized using primers sdAb-Not-R (5' CCAGCGGCCGCTSWGGAGACRGTGACCWGGGTCC 3') and RevertAid kit (Thermo Scientific, Bratislava, Slovakia) was exactly performed as described in our earlier publication (Comor et al., 2017). Thereafter, 500 ng of cDNA was used to amplify the VHH region by PCR using degenerated primers: NB-F-GCGGCCCAGCCGGCCGCCSAGGTGSAGGTSSWGSMGTC and NB-R-AAAGGCCCCCGAGGCCGATSWGGAGACRGTGACCWGGGTCC. The underlined sequence is *Sfi*I site. The cycling conditions were 95°C–2 min and 25 cycles of 95 °C–20 s, 56 °C–30 s and 68 °C–1 min. The reason to use 25 cycles in the PCR reaction was to retain diversity of VHH clones as opposed to amplification of a single species of VHH sequence during the typical 40 cycles. The amplicon of VHH is presented below.

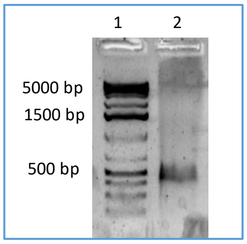

Amplicon of the VHH. *Lane 1 1kb ladder. Lane 2. Amplified VHH*

The amplified VHH were digested with restriction enzyme *Sfi*I (Thermo Scientific Bratislava, Slovakia) at 50°C for 1 h and ligated into *Sfi*I-digested phagemid pJB12 (the phagemid map is shown below in the figure). The purified (Macherey-Nagel, Germany) ligation mix was used to perform 9 electroporations in *E. coli* XL-1 blue (New England Biolabs, Germany) using the preset method in Gene Pulser X cell (Biorad, UK) as described previously (Comor et al., 2017). After incubating the transformed *E. coli* for 1 h in 1 mL SOC medium (New England Biolabs, UK), the culture was plated on 5 LB agar (Sigma Aldrich) plates (diameter 18 cm) supplemented with tetracycline (50 µg/mL, Duchefa Biochemie BV) and chloramphenicol (50 µg/mL, Duchefa Biochemie BV). Transformants growing on LB plates after overnight incubation at 37 °C were scraped. This is designated as VHH-*E. coli* library and stored at – 80 °C in the form of 5 mL aliquots (LB medium: glycerol (MikroChem) (1:1 V/V).

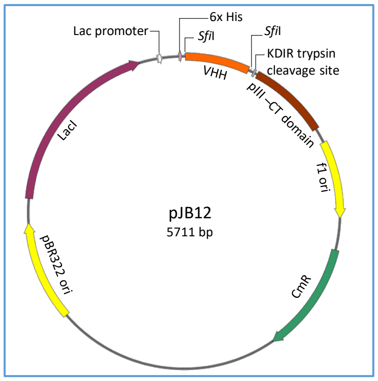

Vector map of pJB12 phagemid used to construct VHH-phage library. *6x His (6 histidine tag), SfiI (SfiI restriction site), KDIR Trypsin cleavage site, pIII-CT domain pf p3, f1 ori (f1 origin of replication), CamR (sequence of chloramphenicol resistance), pBR322 ori (pBR322 origin of replication), LacI (Lac repressor), and Lac promotor region are marked.*

The VHH-*E. coli* library was checked for diversity by sequencing 20 clones. In short, randomly picked *E. coli* colonies were subjected for DNA isolation (heat-shock method) and the VHH region was amplified as described above and sequenced with Sanger sequencing method. The sequences were aligned by Geneious pro 9.1 software and the distance matrix (**shown below in the figure**).

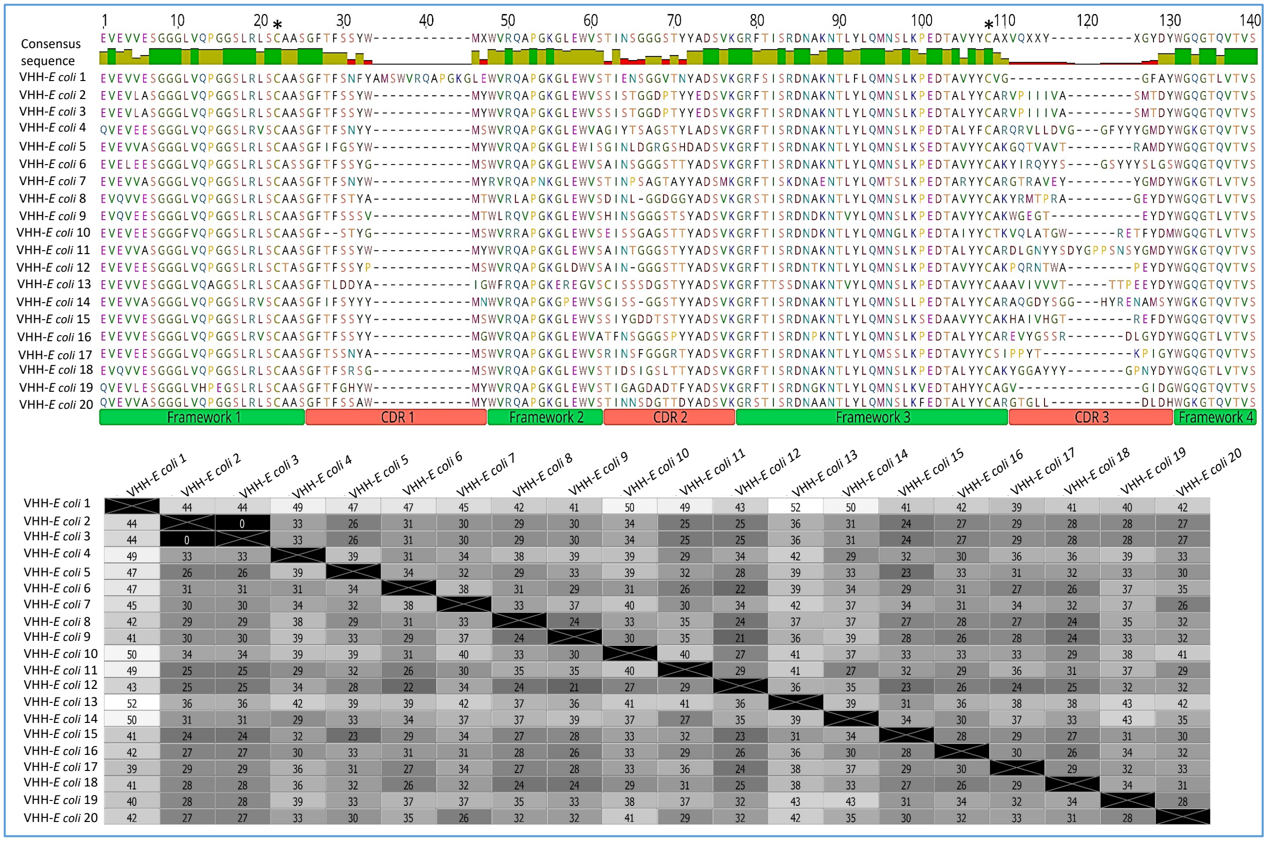

Upper panel *- amino acid sequences of 20 clones randomly chosen from VHH-E. coli library aligned to designate framework and CDRs regions. Two cysteine residues (C22 and C108) potentially forming a disulfide bond are marked with asterisks. Consensus sequence denotes the similarity of residues within the framework region 1–4 and variation in CDR regions: 1–3.* ***Lower panel*** *- Distance matrix plot for the aligned sequences. Each box is mentioned with the number of amino acid residues differing in clone comparison. Various shades of gray correspond to the difference in residues, where in the white color represents maximum difference and black represents no difference.*

The VHH-*E. coli* library (initial OD600 of 0.1) was amplified in 800 mL of the 2xTY medium (16 g/l tryptone, 10 g/l yeast extract, and 5 g/l NaCl; pH = 7.0) supplemented with tetracycline (50 µg/mL), chloramphenicol (50 µg/mL), and glucose (4%) to obtain the final OD600 of 0.5. The amplified VHH-E. coli library was superinfected with VCSM13 helper phages (Agilent, USA, MOI = 20 phages/E. coli, at 37°C for 1 h, static conditions). The superinfected library was incubated at 37°C for 30 min, followed by centrifugation at 3,500 × g to remove unbound phages. The pellet was resuspended in a new 2xTY medium supplemented with 0.1% glucose, tetracycline (50 µg/mL), chloramphenicol (50 µg/mL), kanamycin (50 µg/mL), and 100 mM IPTG and incubated for 16 h at 37°C. The escaped phages were precipitated with PEG-NaCl (20%

polyethylene glycol and 2.5 M NaCl). The precipitated phages were resuspended in a phage dilution buffer (10 mM Tris-HCl, 20 mM NaCl, 2 mM EDTA; pH = 7.3) and enumerated by spectrophotometry using online calculator (Phage Concentration Calculator, 2012). All the chemicals used to perform phage display were procured from Sigma-Aldrich (unless stated otherwise), and the antibiotics were purchased from Duchefa Biochemie BV, Netherlands. These purified phages are designated as the VHH-phage library (**Phage particle with VHH on PIII protein is presented below in figure**).

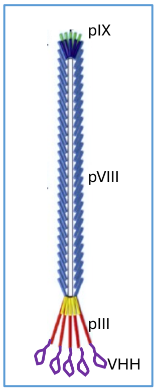

Packaged phage particle showing VHH on pIII protein. *Other units (pIX and pVIII) are shown.*

The Panning: Isolation of VHH-phages having affinity to RBD. 3.9 x 10^{11} phages from the VHH-phage library (library was produced during our previous study (Hruskovicova et al., 2022)) were added in 6 empty wells of Pierce Nickel-coated 96-well plates (Thermo Fisher Scientific, USA) for 1 h at 37°C (pre-adsorption) to eliminate potential nickel and plastic binding phages. After 1 h, the content of the wells was transferred to the wells already blocked with 5% bovine serum albumin for 1 h. The content of the well was then subjected to first round of panning against recombinant receptor binding domain (RBD). In brief, 300 ng of r-RBD was coated in the wells of a nickel plate for 1 h at room temperature. Wells were washed once with PBS and 1% of BSA resuspended in PBS was added to each well for 30 min. The wells were washed once with PBS and the VHH-phage library (supernatant retrieved from pre-adsorption) was incubated on r-RBD-coated wells for 1 h with orbital shaking (100 RPM) at 37°C. Stringent washings (15 times) were performed with Tris-buffered saline containing 0.1% Tween 20 (TTBS) containing 20 mM imidazole (pH 7.2) to eliminate non-specific binders. Elution of specific phages was done in 200 µL of 250mM imidazole in TTBS (pH 7.2). Eluted phages were trypsinized (125 µg/mL of trypsin gold, Promega, USA) for 10 min at 37°C. Immediately, an equal volume of 1% BSA in PBS was added to neutralize the activity of trypsin.

Trypsinized phages (eluate from panning) were amplified by infecting E. coli XL-1 blue (OD600 ~0.5) grown in 10 mL of 2xTY medium supplemented with 4% glucose, tetracycline (50 µg/mL; Duchefa Biochemie BV, Germany), and chloramphenicol (50 µg/mL; Duchefa Biochemie BV) for 1 h at 37°C (static conditions). The culture was centrifuged, supernatant was discarded and the pellet was resuspended in fresh 2xTY medium supplemented glucose, tetracycline, and chloramphenicol as above. Culture was incubated overnight in shaker incubator (37 °C, 300 RPM). The overnight grown culture (1mL) was inoculate into 50 mL of 2xTY medium (supplements same as before) and incubated to attain the OD600 ~0.4. Thereafter, the culture was superinfected with VCSM13 helper phages (Agilent, USA, MOI = 20 phages/E. coli, at 37 °C for 1 h, static

conditions). The incubation was continued for 16 h on shaker-incubator (200 RPM) at 25 °C and the escaped phages were harvested by precipitation by adding 25% V/V of PEG-NaCl [20% polyethylene glycol (Sigma Aldrich, Germany), 2.5 M NaCl (Sigma Aldrich)]. Final resuspension of precipitated phages was performed in phage dilution buffer (10 mM Tris-HCl pH 7.5, 20 mM NaCl, and 2 mM EDTA, Sigma Aldrich) and enumeration was done by spectrophotometry using the formula [(A269–A320 * $6x10^{16}$)/(number of bases per virion)] = phages per mL.

Phages enumerated above were used in the second round of panning against r-RBD keeping all condition exactly described above, however the number of phages used in panning were 3.9×10^{10} and concentration of r-RBD used was only 30 ng/well. The phages obtained from second round were used in third round. Phages obtained from third round were stored in glycerol stock in -80°C.

Supplementary information 3. Panning—Isolation of C7C peptides

$1x10^{12}$ phages from the Ph.D.-C7C library (New England Biolabs, USA) were pre-adsorbed to remove nickel and plastic binder as described above. The pre-adsorbed library was incubated with RBD coated in Pierce nickel-coated 96-well plates for 1 hr at room temperature with constant shaking. Wells were then washed 15 times with the same washing buffer (PBS containing 0.05% Tween20), and phages were eluted with imidazole as described above. For detailed protocol, we recommend using the online manual for Ph.D.-C7C from New England Biolabs: https://www.neb.com/en/products/e8120-phd-c7c-phage-display-peptide-library-kit. After each elution, the number of phages was calculated with the plate titration method, and the rest of the eluate was subjected to amplification of phages in *E. coli* ER2738 (New England Biolabs) for 4.5 hrs at 37°C. Amplified phages were precipitated from *E. coli* culture with polyethylene glycol (PEG)/NaCl precipitation as per the manufacturer's instructions (New England Biolabs). In short, amplified phages were mixed with 20 % PEG 8000 in 2.5 M NaCl and allowed to precipitate at 4 °C overnight. The PEG precipitation was spin (12,000 × g for 15 min at 4°C). The pellet of amplified phages was resuspended in 1 mL of PBS and spun (15,000 × *g* for 5 min at 4°C) to remove residual cells. The phage-containing supernatant was reprecipitated with 1/6th volume of 20 % PEG 8000 in 2.5 M NaCl for 1 hr at 4°C. The PEG precipitation was centrifuged (15,000 × *g* for 15 min at 4°C), and the phage pellet was resuspended in 200 µL of PBS. Such purified phages were used for the next round of panning. A total of 3 rounds of panning were performed in this study. Phages obtained from the third round were enumerated by spectrophotometry using the formula $[(A_{269}–A_{320}\ ^{*}\ 6x10^{16})/(\text{number of bases per virion})]$ = phages per mL. Phage pool was stored in glycerol stock at -80°C.

Supplementary information 4. Phage ELISA

Confirmation of binding of phages after 3rd round of panning to spike protein. Phage ELISA was performed as described in our earlier publication (Mertinkova et al., 2021). Briefly, either 1 µg of recombinant spike was coated on a 96-well plate for 16 h at 4 °C. A quick wash with TTBS (Tris buffer saline containing 0.05% Tween 20, pH 7.2) was performed before blocking the wells with 5% BSA in TTBS (1 h at room temperature). Thereafter, the phage pool eluted from the 3rd round of panning (1 x 10^{11} particles either from the VHH-phage or C7C phage library) resuspended in 100 µL of TBS containing 1% BSA was added, and the plate was incubated for 1 h at room temperature (orbital shaking 100 RPM). Three washes were performed, and mouse anti-M13 antibody (1:1000 in TTBS and 1% BSA) was added (1 h at room temperature, orbital shaking). Again, three washings were performed, and horseradish peroxidase (HRP) conjugated Protein A/G (1:10 000 in TTBS and 1% BSA, Thermo Fisher Scientific) was added in the wells for 1 h. Lastly, the chromogenic reaction was developed in 25 min by 1-Step™ Ultra TMB-substrate (Thermo Fisher Scientific). The reaction was stopped with 2 M H_2SO_4 (Sigma), and the absorbance was measured at 450 nm (Victor, Multilabel plate reader, Parkin Elmer). For the input control, wells coated with 3.2 x 1010 VCSM13 helper phage were incubated with the aforementioned antibodies and reagents. Whereas, antigen-coated wells devoid of phage incubation served as negative control. The entire assay was performed in triplicate, and the absorbance value (mean ± SE) was compared for statistical significance ($p < 0.05$) using one-way ANOVA and Bonferroni's post hoc test on Prism v.9 (GraphPad Software, Inc., San Diego, USA).

Supplementary information 5. Isolation of individual VHH-phage and C7C-phage clones

VHH-phage. A phage pool from the 3rd round of panning was serially diluted (10-fold) in 30 µL of 2xTY medium supplemented with 4% glucose, tetracycline (50 µg/mL), and chloramphenicol (50 µg/mL). The diluted phages, varying from 4.8 $x10^{11}$ to 4.8, were used to infect 100 µL of *E. coli* XL-1 blue (OD_{600} ~0.4) for 1 h at 37°C (static conditions) and spread plated on a 2xTY agar plate (supplements same as above). 96 solitary colonies were randomly picked and propagated into a 96-deep well plate (VWR International s.r.o, Bratislava, Slovakia) containing 1 mL of 2xTY medium (supplements same as above). Propagation was performed at 37°C (shaking at 200 RPM) and 25 µL of overnight culture was inoculated into 1 mL of 2xTY medium until the final OD_{600} reached 0.4. Thereafter, the culture was superinfected with VCSM13 helper phage (MOI = 20 phages/*E. coli*) for 1 hr. The plate was centrifuged at 6000 RPM for 20 min, and the supernatant was discarded. 2xTY medium supplemented with 0.1% glucose, tetracycline (50 µg/mL), chloramphenicol (50 µg/mL), kanamycin (50 µg/mL), and 100 mM IPTG was added in each well, and the plate was incubated overnight at 30°C with constant shaking to escape the phages. The next day the plate was centrifuged (6000 RPM for 20 min), the supernatant was collected and transferred to a new plate, which was centrifuged again with the same speed and time as stated above. Supernatant containing phages was collected carefully in a new plate (to avoid any cell debris), and 150 µL of 20% PEG 8000 in 2.5 M NaCl was added, and the plate was incubated for 1 hr at 4°C. The plate was centrifuged (10,000 × *g* for 45 min at 4°C), and the supernatant was discarded carefully. The phage pellet was resuspended in 200 µL of PBS. Such purified phages were mixed with glycerol (1:1 MikroChem), divided into aliquots, and stored at -80°C for further use (phage ELISA, extraction of DNA, etc.).

C7C-phage. A phage pool from the 3rd round of panning was serially diluted (10-fold) in 30 µL of 2xTY medium, incubated with 170 µL of *E.coli* (growing in log phase) for 1 h and then plated on LB agar plates containing 1 mM IPTG (Fermentas, Slovakia), 1 mM X-gal (Sigma, USA). Plates were incubated at 37°C overnight, and 96 well-separated phage plaques were randomly picked and propagated in LB medium containing *E.coli* growing in log phase in a 96-deep well plate as described above. Please note that the C7C-phage library does not need helperphage for packaging and escape. After overnight incubation, the escaped phages in the supernatant were separated, precipitated, aliquoted and stored at -80°C exactly as described above.

Supplementary information 6. Phage ELISA for individual clones

Phage ELISA was performed to screen the clones for their ability to bind spike protein. In short, 300 ng (2.13 pM) of spike protein was coated in each well of the 96-well plate. After blocking the wells with 5% BSA in TTBS, 10 µL of individual phage clone (prepared above, Supplementary information 5) resuspended in 90 µL of TTBS was added and incubated for 1 h at 37°C. Three washings with TTBS were performed, and mouse anti-M13 antibody (1:1000 in TTBS and 1% BSA) was added to each well, incubated for 1 h, and the wells were washed three times with TTBS. Protein A/G-HRP (1:10 000 in TTBS and 1% BSA) was added, incubated for 1 h, and the wells were washed three times. The chromogenic reaction was developed by 1-Step Ultra TMB-substrate. The reaction was stopped with 2 M H_2SO_4 and the absorbance was recorded at 450 nm. For the positive (input) control, wells were coated with 3.2 x 10^{11} phages from the 3rd round of panning, and for the negative control, the phage clones were omitted from the assay. The absorbance of chromogenic reaction above 0.5 was considered as the threshold for interaction between phages and spike protein.

Supplementary information 7. Isolation of phage ssDNA and sequencing.

Genomic ssDNA was isolated from individual phage clone. In short, an aliquot of phage clone (prepared in supplementary information 4) was heated at 98 °C for 10 minutes. The DNA released in supernatant was used directly for PCR.

Primers used for PCR were as follows:

VHH-phages: pJB12F - ATGAAATACCTATTGCCTACGGCAG & pJB12R - CATAATCAAAATCACCGGAACCAGAG

C7C-phages: M13KEF - TCGCAATTCCTTTAGTGGTACCTTTC & M13KER - GCATTCCACAGACAGCCCTCA

The reaction mix for both sets of primers was as follows: PCR-grade water – 38.1 µL; 10X Taq Polymerase Buffer – 5 µL; 12.5 mM dNTP – 0.5 µL; 10 µM forward primer – 0.6 µL; 10 µM reverse primer – 0.6 µL; 5 units/µL Taq polymerase – 0.2 µL; DNA – 5 µL.

Cycling condition for both sets of primers was*:* 94 °C – 2 min; 35 x [94 °C – 30 sec, 60 °C – 30 sec, 72 °C – 1 min 45 sec]; 72 °C – 10 min; 4 °C – forever.

Amplicons were resolved on 1% borax gel for 90 min at 140 V. Bands were cut off and purified by column purification (Macherey-Nagel, Germany). Purified amplicons were sequenced by Sanger sequencing, with the same sets of primers using BigDye Terminator v 3.1 Cycle Sequencing Kit (ThermoFisher scientific, USA). The amino acid sequences (in silico translated DNA sequences) of each clone were aligned in silico and clustered by sequence homology (Geneious pro 9.1, Biomatters).

Supplementary information 8. Production of soluble VHH and C7C

Production of soluble VHH in *E. coli*. The VHH fragment was amplified using following primer set, wherein the underlined sequence is *Sfi*I site.

NB-F-GC<u>GGCCCAGCCGGCC</u>GCCSAGGTGSAGGTSSWGSMGTC

NB-R-AAA<u>GGCCCCCGAGGCC</u>GATSWGGAGACRGTGACCWGGGTCC

The DNA isolated from phage clones was used for amplification. The PCR conditions were – 95 °C–2 min and 35 cycles of 95 °C–20 s, 56 °C–30 s and 68 °C–1 min. The resulting ~500 bp amplicon (see below panel A) was digested with restriction enzyme *Sfi*I (Thermo Fisher Scientific) at 50°C for 1 h and ligated into *SfiI* digested in-house modified vector pQE30-UA-mCherry-GFP containing *Sfi*I sites (see below panel B).

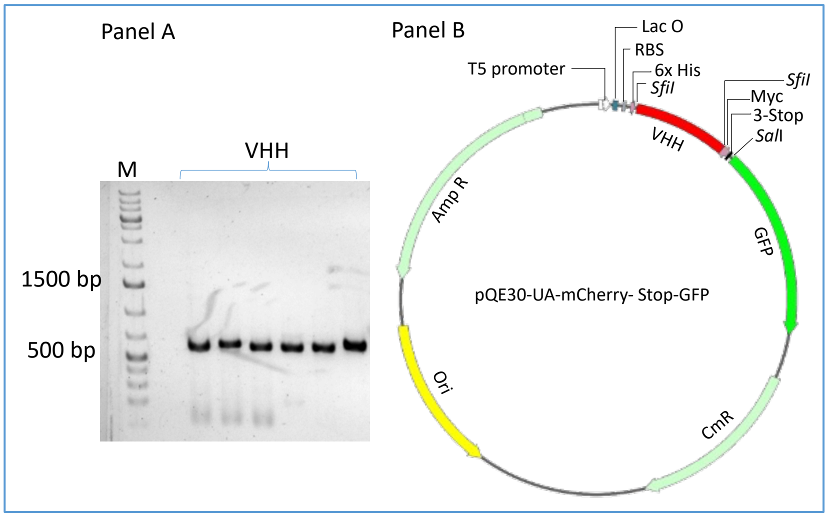

Panel A. *VHH gene fragment (~500 bp) resolved on agarose gel. M- 1Kb DNA ladder, VHH – amplicons of VHH.* ***Panel B.*** *Vector map of plasmid pQE30-UA-mCherry-GFP with SfiI sites used to ligate VHH gene fragment. T5 promoter, LacO (Lac operator), RBS (ribosomal binding site), 6x His (6 histidine tag), BamHI (BamHI restriction site), SfiI (SfiI restriction site), SalI (SalI restriction site), GFP (GFP sequence), CmR (sequence of chloramphenicol resistance), Ori (origin of replication), and Amp R (sequence of ampicillin resistance) are marked.*

The ligation mix was column purified, and the purified ligation mix (100 ng) was electroporated into *E. coli* SHuffle (New England Biolabs, UK) using Gene Pulser X cell™ (BioRad). The transformed mix was plated on LB agar plates supplemented with carbenicillin (100 µg/mL) (Duchefa Biochemie) and incubated at 30°C overnight. A single colony for each VHH clone was picked and propagated into 50 mL of TB medium [tryptone 12 g/L (Duchefa Biochemie), yeast extract 24 g/L (Duchefa Biochemie), glycerol 0.6% (MikroChem), 100 mL/L of $Na_2HPO_4.12H_2O$ (25 mM, Sigma Aldrich), KH_2PO_4 (25 mM, Sigma Aldrich) and 20 mL/L glucose (Duchefa Biochemie)] supplemented with carbenicillin (100 µg/mL, Duchefa Biochemie) for 16 h at 30°C (shaking incubation 250 RPM). Induction of VHH expression was performed in 50 mL TB medium (supplemented with carbenicillin and 0.5 mM IPTG) at 30°C for 3 h (shaking at 250 RPM) followed by 22°C for 16 h (shaking at 250 RPM). The induced bacterial pellet was lysed during 4 freeze-thaw cycles in the presence of denaturating lysis buffer (50 mM $NaH_2PO_4.2H_2O$, 300 mM NaCl, 8M Urea, 10% glycerol, 10 mM imidazole, pH 8). Additionally, 10 rounds of sonication (45-s burst at 75 Hz, 1 min pause) were performed to ensure complete *E. coli* lysis. Lastly, the lysate was purified by nickel affinity chromatography using Ni-

NTA agarose beads (Jena Bioscience, Germany) and size exclusion chromatography on Äkta purifier (GE Health care) using Sephadex G25 (30 mL column, in-house prepared, see below the chromatograph) as described elsewhere (Kanova et al., 2018; Mertinkova et al., 2020). The purified VHH was assessed for its quality on SDS-PAGE before its storage at -80 °C.

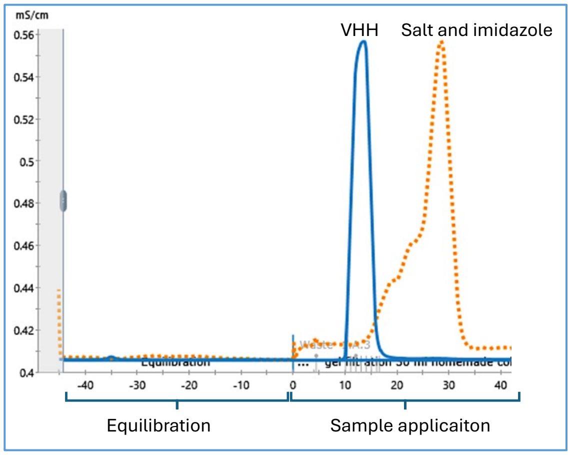

Size-exclusion chromatography (SEC) of the VHH performed on Sephadex G25 (30 mL column) against ammonium bicarbonate buffer (25 mM, 7.8 pH). The equilibration step – 45 ml (1.5 column volume) of the ammonium bicarbonate buffer passed through the column to equilibrate it, followed by the sample application/elution step (isocratic elution). The large molecule (like VHH) elutes first (blue peak), separated from small molecules like salt, urea, imidazole, etc. (orange dotted peak).

Production of C7C peptide. The 7-mer cyclic from selected phage clones were produced in the *E. coli* Shuffle express (New England Biolabs) expression system. PCR conditions, digestion, and ligation into the pQE-30-UA-mCherry-STOP expression vector, *E. coli* transformation, clonal selection, protein overexpression, purification, and quality control were exactly as per our previous publication (Petrouskova et al., 2025). Please note that overexpressed the C7C peptides contains N-terminal tag (6xHis-28aa-GGGGS, detail sequence is in our publication (Petrouskova et al., 2025)). C7C with N-terminal tag was used in ELISA to assess binding with SARS-CoV-2 virion. After confirmation of the binding, selected clones were subjected to cleavage of the N-terminal tag with bovine enterokinase (GenScript Biotech) as described by us (Petrouskova et al., 2025). The purity of the cleaved peptides was checked by SDS-PAGE and MALDI-TOF-MS, and peptides were used in virus neutralization tests (pseudovirus neutralization and plaque reduction neutralization test), cell toxicity assay, hemocompatibility assay, and crossing of peptide across the blood-brain barrier in vitro.

Supplementary information 9. Pseudovirus (virus like particle, VLP) titration and their pseudovirus neutralization assay

VLP titration. Dilution plate (see below schematic presentation of the plate): The blue wells in the periphery indicate wells in which 250 µL of sterilized water was added (no samples). The maroon wells (B11 – G11) show the cell control (CC) wells, in which only DMEM (150 µL) was added. Yellow wells were used for the initial dilution of VLP (1:10) and wells in column 3 to 10 were used for serial dilution as follows: in B2 to G2 well, 135 µL of complete DMEM was added, while in remaining wells (B3 – G11) 100 µL of complete DMEM was added. 15 µL of pseudotyped virus was added in each well in column 2 (wells B2 – G2). Mixed six times. 50 µL from the wells B2 – G2 were transferred to wells B3 – G3 with a multi-channel pipette, mixed six times and 50 µL were transferred to B4 – G4 wells. Dilution was repeated till the B10–G10 wells. Finally, 50 µL of the solution from each well from column 10 was discarded.

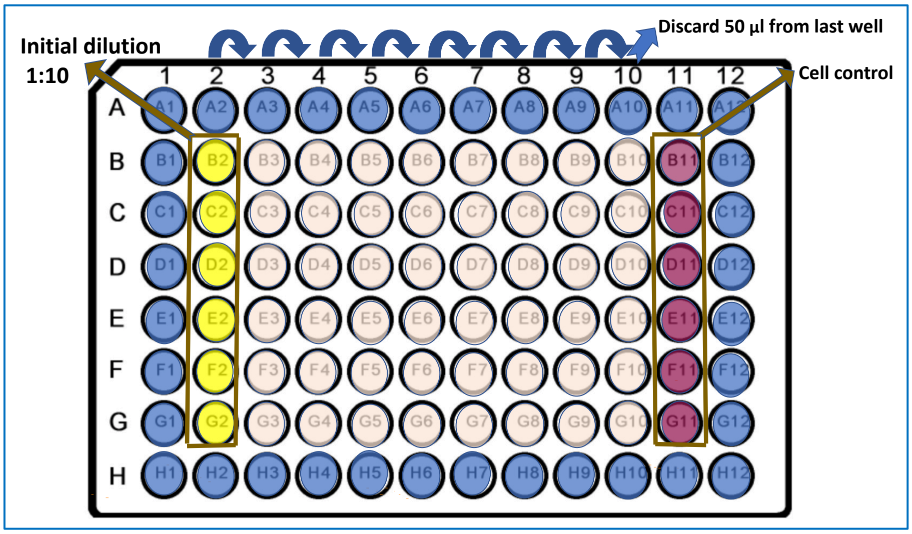

Blue wells – sterilized water was added (no samples); wells in yellow colour used for initial dilution of VLP, wells in cream color - serial dilutions of the VLP, wells in maroon colour – cell control (no virus added).

Human embryonic kidney cells 293/17 (HEK293/17, passage 11) expressing high levels of ACE2 (gifted by Axon Neuroscience, Bratislava) were cultured in a 96-well plate (TPP, Thermo Scientific; 20,000 cells/well) containing 150 µl of DMEM, high glucose, GlutaMAX (Gibco, Thermo Scientific) supplemented with L-glutamine (2 mM, Thermo Scientific), 10% fetal bovine serum (Thermo Scientific), 2% HEPES, and 1x penicillin–streptomycin (Jana Biosciences) at 37°C in 5% CO_2.

Diluted VLPs were transferred to a HEK293T/17 cell culture plate and incubated at 37°C and 5% CO_2 for 48 h. Infected HEK293T/17 cells were lysed for 5 min at room temperature with 20 µl of lysis buffer, a component of the luciferase assay system kit (Promega, USA). The lysate was transferred to a 96-well plate (white-walled and bottom wells; CELLSTAR, USA), and 100 µl of the luciferase assay reagent was added before measuring the luminescence on Cytation 7 (Biotek, USA) with the parameters: integration time: 10 s, read height: 5.4 mm and gain:240. Values obtained from Cytation 7 were pasted in the Excel template provided in our previous publication (Hruskovicova et al., 2022) **.** Please note that this template was adopted from the recently published article (Nie et al., 2020) and the calculation of $TCID_{50}$/mL was performed according to the Reed-Muench method(Matumoto, 1949).

Pseudovirus neutralization assay. Dilution plate (see below schematic presentation of the plate): The blue wells in the periphery indicate wells in which 250 µL of sterilized water was added (no samples). The dark-brown wells (B2 – G2) show the cell control wells, in which only DMEM (150 µL) was added. Red wells (B3 – G3) indicate virus control wells, in which 100 µL of DMEM and 50 µL of VLP were added. Neutralization: Columns 4 and 5 are for one sample (duplicate), thus 4 samples can be tested in one plate as shown in figure. In wells C4 – G11, 100 µL of DMEM was added (not in row B, B4-B11). The VHH or C7C peptides were serially diluted as follows in dilution plate: 1 µg of the VHH or peptide (in 7.5 µL) was mixed with 142.5 µL of DMEM in wells B4 and B5. Content was mixed six times. Fifty microliters from each well were transferred to C4 and C5, mixed six times, and 50 µL from C4 and C5 were transferred to D4 and D5. Dilution was continued till G4 and G5 wells and 50 µL from those wells were discarded. 50 µL of VLP (diluted to achieve 400-500 TCID50/well) were added to each well in duplicate. The plate was incubated for 3 hrs at room temperature. Transfer of VHH/C7C and VLP mix to cells: HEK293T cells (20,000 cells/well) were cultured in a 96-well plate (except peripheral wells) in complete DMEM medium overnight. The medium was removed carefully, and the content of each well from the dilution plate was transferred to the culture plate. The plate was incubated at 37 °C, 5% CO_2, for 48 h. After incubation, wells were checked under the microscope and subjected to luciferase assay.

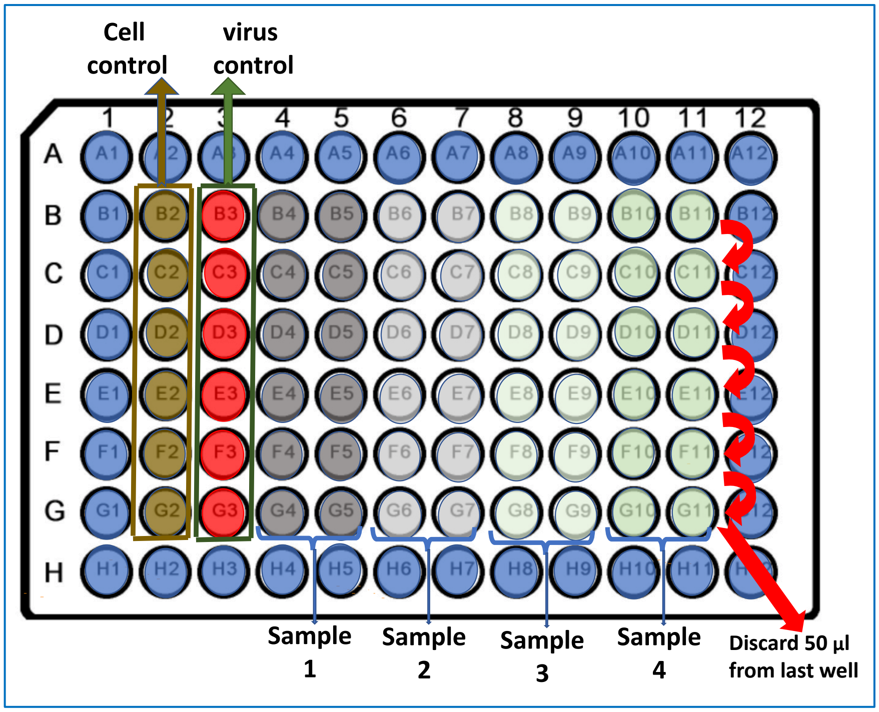

Supplementary information 10. Plaque reduction neutralization test (PRNT)

VERO E6 cells were cultivated in 75-cm^2 flasks in Minimum Essential Medium (MEM; Serana, Germany) supplemented with 5% FBS and 2mM L-glutamine at 37°C and 5% CO_2. After receiving 80% of confluence, cells were infected with 10^6 plaque-forming units (PFU) of SARS-CoV-2 (lineage BA.5, Omicron variant) and incubated for 48 h at 37°C and 5% CO_2. Cells were disrupted with two freeze-thaw cycles and then centrifuged at 4000 *x g* for 10 min. The virus-containing supernatant was concentrated on VivaSpin columns (100 kDa; Cytiva, USA) and kept in aliquots at -80°C until used.

Virus titration. VERO E6 cells were seeded in a 24-well plate ($8x10^4$ cells/well) and cultivated overnight under the same conditions as described before. One aliquot of the virus (live virus lineage BA.5, Omicron) was serially ten-fold diluted (20:200 ratio; 200 µL in each dilution) in complete MEM medium. Each dilution was added to cells and incubated for 1 h at 37°C and 5% CO_2. The medium containing the virus was removed, and cells were overlaid with 1.5% carboxymethyl cellulose (CMC; Merck, Germany) in 2X complete MEM (1:1 [v/v]), following the 48 h of incubation at 37°C and 5% CO_2. The cells were fixed with 500 µL of 8% formaldehyde for 30 min. Wells were washed with water, and plaques were visualized by staining with 0.5% crystal violet for 15 min at room temperature (VWR, USA). Wells were washed with water, the number of plaques was counted, and virus titre was expressed as plaque-forming units (PFU) per mL. The assay was performed in triplicate.

The plaque-reduction neutralization test (PRNT). VERO E6 cells were seeded in a 24-well plate ($8x10^4$ cells/well) and cultivated overnight under the same conditions as described before. 100 plaque-forming units (PFU) of live (strain lineage BA.5, Omicron) were pre-incubated with either 1. VHH_{E12}, or 2. $C7C_{18}$, or 3. heat-inactivated (56°C for 30 min) hyperimmune serum from an individual vaccinated against SARS-CoV-2, or 4. PBS (VC, Virus Control). Untreated cells were also kept in assay (CC, cell control). VHH and C7C were used in 5 dilutions (final concentrations 3.125, 0.78, 0.195, 0.045, and 0.010 µg/mL), while human sera were 4-fold diluted. After pre-incubation (90 min in a cell culture incubator at 37°C), all treatments were transferred to the cells and incubated for 2 h at 37 °C. Then, carboxymethylcellulose (1.5%) in the cell-culture medium was overlaid and incubated for 3 days. Cells were fixed with 4% formaldehyde in PBS for 10 min, stained with crystal violet, and PFUs were counted. % reduction in plaque formation was calculated against plaques in VC – virus control.

Supplementary information 11. Production of VHH and C7C fused with Angiopep-2

Strategy of fusion of Angiopep-2.

VHH. In the case of VHH, the His-tag and Angiopep-2 were fused at the N-terminus to keep the CDR3 (antigen-binding region) free. Details are presented in the figure below.

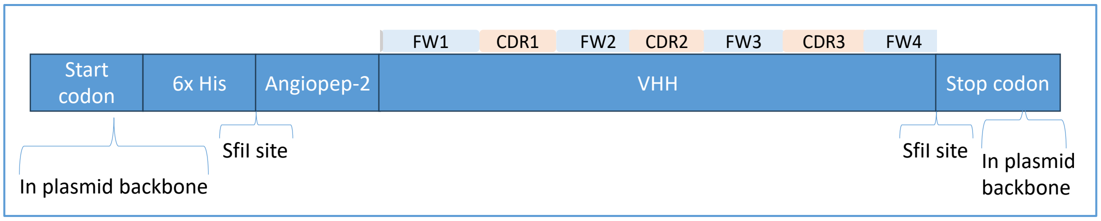

The VHH was amplified with primers carrying overhangs as follows – the forward primer had an SfiI site, followed by the coding sequence for Angiopep-2 and a complementary sequence of VHH (FW1). The reverse primer contained an overhang for the SfiI site. The start codon, 6x His tag, and stop codon are built in the expression vector.

The 6x His tag and Angiopep-2 were kept at the N-terminus of the VHH, as the major antigen binding (CDR3) is present at the C-terminus. Thus, the C-terminus of VHH remained free from any steric hindrance that would be caused by the adjacent tag.

C7C. In the case of C7C, the Angiopep-2 was kept at the C-terminus, as the antigen-binding region is at the N-terminus.

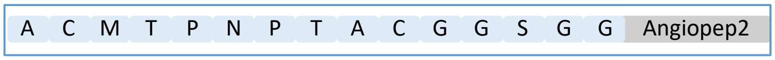

The binding site in the C7C peptide is encompassed by two cysteine residues. The C7C peptide is displayed on the phage fused with the pIII protein, with alanine at the N-terminus, followed by cysteine, 7 amino acids, cysteine, and the linker glycine-serine linker (GGSGG). The New England Biolabs, manufacturer of the phage-display library, has recommended maintaining the N-terminal alanine and C-terminal glycine-serine linker in the synthetic peptide. The sequence of Angiopep-2 was thus after the glycine-serine linker.

Production of VHH-angiopep-2. The VHH fragment was amplified from *E. coli* carrying clone VHH_{E12} using the following primers, wherein the underlined sequence is *Sfi*I site and the bold sequence encodes angiopep-2.

NB-Angio-F
GCGGCCCAGCCGGCC**ACCACCTTTTTTTATGGCGGCAGCCGCGGCAAACGCAACAACTTTAAAACCGAAGAATAT**SAGGTGSAGSTSSWGSMGTC
NB-R
AAAGGCCCCCGAGGCCGATSWGGAGACRGTGACCWGGGTCC

The PCR conditions were – 95 °C–2 min and 35 cycles of 95 °C–20 s, 60 °C–30 s and 68 °C–1 min.

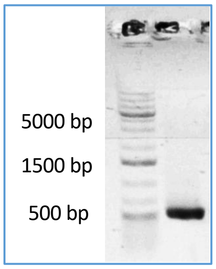

PCR product of VHH with overhang at 5' encoding angiopep-2

The resulting ~560 bp amplicon was digested with restriction enzyme *Sfi*I (Thermo Fisher Scientific) at 50°C for 1 h and ligated into *SfiI-digested* in-house modified vector pQE30-UA-mCherry-GFP containing *Sfi*I sites (the vector is presented in **supplementary information 8**). The ligation, electroporation in *E. coli* Shuffle clonal selection, overexpression of protein, purification using Ni-NTA agarose beads followed by gel filtration, and quality control with SDS-PAGE were performed exactly as described in **supplementary information 8**.

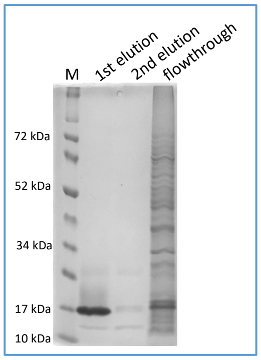

Results of SDS-PAGE of the purified $VHH_{E12\text{-}Ang2}$. The protein was purified with Ni-NTA chromatography in which protein was eluted with imidazole in two elution steps – 1st and 2nd. Most of the protein was eluted in 1st elution. Flowthrough indicates the E.coli lysate after capture of the protein. M – molecular weight marker

$C7C_{18}$-angiopep-2 and $C7C_{18}$ peptides. ACMTPNPTACGGSGG**TFFYGGSRGKRNNFKTEEY*** and ACMTPNPTACGGSGG* were purchased (MedChemExpress, USA). The underlined sequence is $C7C_{18}$ followed by the GGSGG linker. The bold sequence is of Angiopep-2. * indicates conjugated peptides with infrared dye (680 nm).

Conjugation of VHH_{E12} and VHH_{E12}-angiopep-2 with infrared dye (680 nm). 1 mg of the purified VHHs resuspended in Phosphate-buffered saline, PBS, pH 7.2 (1 mg/mL) was mixed with IRDye 680LT peptide labelling reagent (Licor bio, USA), total volume was adjusted to 500 µl by adding PBS, as recommended by the manufacturer and incubated for 3 hrs in the dark with constant shaking. The unbound dye was removed using an MWCO spin column (3 kDa cutoff, Cytiva, USA), and conjugated VHHs were washed twice in the same column with 500 µl of PBS.

Supplementary information 12. Virus neutralization test of the Angiopep-2 conjugated VHH and C7C

Virus neutralization test for VHH and C7C conjugated with Angiopep-2. The ability to neutralize the virus after conjugation with iAngiopep-2 was tested with a virus neutralization test using live virus (lineage BA.5, Omicron) as described in supplementary information 10. Test molecules were mixed with 50 PFU of virus (final volume 500 µL), incubated for 90 min, and then allowed to infect the cells. Concentration of the test molecules used were as follows: for the $C7C_{18}$ and $VHH_{E12,}$ EC_{50} concentration was used i.e. 0.01 and 0.045 µg/ml, respectively. For $C7C_{18}$-angiopep-2 and VHH_{E12}-angiopep-2, 0.024 µg/ml and 0.050 µg/ml were used to maintain equimolar ratio to their unfused molecules. 50 µL of heat-inactivated hyperimmune serum was also incubated with virus, which served as serum control. Virus (50 PFU) without preincubation with test compounds served as virus control (VC), and the cells without any treatment served as cell control (CC). Incubation of the cells, fixation, and staining were performed exactly as described before in supplementary information 10. The assay was performed in triplicate.

Supplementary information 13. Crossing of *in vitro* blood-brain barrier

Cultivation of human brain microvascular endothelial cells (BMECs). Cells were cultured exactly as described in our earlier publication(Hruskovicova et al., 2022). Briefly, 1 x 10^6 BMEC (cell line D3; Merck Millipore, USA) were seeded in a collagen-I (Corning, USA) coated T-75 cell culture flask (Sarstedt, Germany) in complete endothelial medium at 37°C, 95% humidity, and 5% CO_2 until confluence. The complete endothelial medium contains DMEM-F12 (Sigma-Aldrich, USA) supplemented with 1.4 µM hydrocortisone (Sigma-Aldrich), 10 ng/mL bFGF (Sigma-Aldrich), 5 mg/mL ascorbic acid (Sigma-Aldrich), 1x penicillin-streptomycin (Jena Bioscience, Germany), 10 mM HEPES (Biowest, France), 2 mM L-glutamine (Life Technologies, USA), and 10% fetal bovine serum (Biowest).

Construction of the in vitro BBB model. 5 x 10^5 BMEC were seeded in the luminal chamber of collagen-I-coated 24-transwell inserts (1 µm pores; cellQuart, Germany, transwell system) containing 600 µL of complete endothelial medium in both luminal and abluminal chambers. The inserts were incubated at 37°C in a humidified CO_2 incubator until a monolayer was formed. The formation of the monolayer was verified by measuring the transendothelial electrical resistance (TEER) between the luminal and abluminal chambers of inserts. In short, TEER was measured by a pair of chopstick electrodes on a volt-ohm meter EVOM (World Precision Instrument, USA). The resistance is inversely proportional to the growth area of the semipermeable membrane (cm^2). The TEER value was calculated using the formula:

$$TEER\ (\Omega/cm^2) = resistance_{insert} - resistance_{blank} x\ growth\ arrea$$

Wherein: Resistance$_{insert}$ *= recorded resistance value between luminal and abluminal chambers of inserts in which the BMEC monolayer is fully grown. Resistance*$_{blank}$ *= recorded resistance value of semipermeable membrane in transwell without cells (only collagen coated). The growth area of the 24-well insert is 0.3 cm².*

When the observed TEER value was ~100 Ω/cm^2, the barrier properties were confirmed by the diffusion of dextran CF770 (Biotium, USA). Briefly, 1 µg of Dextran CF770 was added to the luminal chamber of inserts, and its passage through the BBB model was recorded by scanning the contents of the abluminal chamber at 800 nm on the Odyssey CLx imaging system at 1, 3, and 5 h. The concentration of Dextran CF770 in the abluminal chamber was determined by a standard curve prepared by a two-fold dilution of 2 µg of Dextran CF770. Barrier properties were confirmed when the amount of Dextran CF770 that passed from the luminal to the abluminal chamber was lower than 5% of the amount added to the luminal chamber. Inserts with confirmed barrier properties (by both TEER and Dextran CF770) were used to test the passing of VHH and C7C molecules with and without Angiopep-2 conjugation.

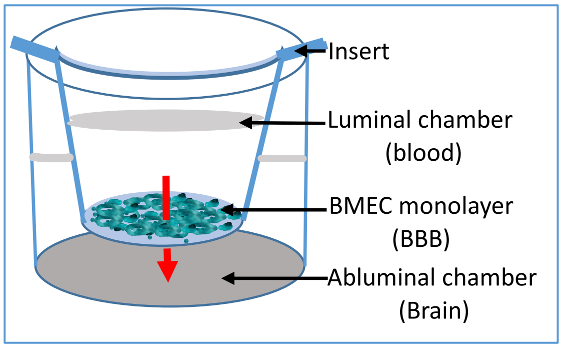

***The BBB model** (transwell system). The luminal chamber represents the bloodstream, the monolayer of BMEC represents the BBB, and the abluminal chamber represents brain tissue. The red arrow shows the way of passing of molecules into the brain.*

Crossing of test molecules through in vitro BBB.

Preparation of standard curves. One microgram of each test compound ($C7C_{18}$-angiopep-2 or $C7C_{18}$ or VHH_{E12} or VHH_{E12}-angiopep-2, all conjugated with infrared dye) or Dextran CF 770 (1 µg) or Angiopep-2-Cy5.5 (1 µg) was mixed with 1.2 mL of complete endothelial medium in 24-well plates and 2-fold serially diluted. Next, the plates were scanned using the Cytation 7 imaging system (BioTek, USA) or Odyssey CLx imaging system using wavelengths of 650/705 nm for IRDye 680LT conjugated VHH and C7C and 770/797 nm for CF770. The measured fluorescence was plotted against the concentrations in each dilution. The concentrations were calculated using a regression curve in GraphPad (https://www.graphpad.com/). ***Translocation assay.*** The *in vitro* BBB model was cultured as described above. One microgram of each test compound enlisted above was added in the upper (luminal) chamber of the BBB model and incubated at 37°C in a cell culture incubator for 1, 3, and 5 h. The crossing of test compounds from the luminal to the abluminal chamber was measured by measuring the fluorescence in the abluminal chamber at 650/705 nm. Fluorescence was measured on the Odyssey CLx imaging system (Li-cor bio, USA). The percentage of passage of the compound was measured by correlating with the standard curve (prepared above). The assay was performed in triplicate.

References:

Comor, L., Dolinska, S., Bhide, K., Pulzova, L., Jimenez-Munguia, I., Bencurova, E., et al. (2017). Joining the in vitro immunization of alpaca lymphocytes and phage display: rapid and cost effective pipeline for sdAb synthesis. *Microb Cell Fact* 16(1), 13. doi: 10.1186/s12934-017-0630-z.

Hruskovicova, J., Bhide, K., Petrouskova, P., Tkacova, Z., Mochnacova, E., Curlik, J., et al. (2022). Engineering the Single Domain Antibodies Targeting Receptor Binding Motifs Within the Domain III of West Nile Virus Envelope Glycoprotein. *Front Microbiol* 13, 801466. doi: 10.3389/fmicb.2022.801466.

Kanova, E., Jimenez-Munguia, I., Majerova, P., Tkacova, Z., Bhide, K., Mertinkova, P., et al. (2018). Deciphering the Interactome of Neisseria meningitidis With Human Brain Microvascular Endothelial Cells. *Front Microbiol* 9, 2294. doi: 10.3389/fmicb.2018.02294.

Matumoto, M. (1949). A note on some points of calculation method of LD50 by Reed and Muench. *Jpn J Exp Med* 20(2), 175-179.

Mertinkova, P., Kulkarni, A., Kanova, E., Bhide, K., Tkacova, Z., and Bhide, M. (2020). A simple and rapid pipeline for identification of receptor-binding sites on the surface proteins of pathogens. *Sci Rep* 10(1), 1163. doi: 10.1038/s41598-020-58305-y.

Mertinkova, P., Mochnacova, E., Bhide, K., Kulkarni, A., Tkacova, Z., Hruskovicova, J., et al. (2021). Development of peptides targeting receptor binding site of the envelope glycoprotein to contain the West Nile virus infection. *Sci Rep* 11(1), 20131. doi: 10.1038/s41598-021-99696-w.

Nie, J., Li, Q., Wu, J., Zhao, C., Hao, H., Liu, H., et al. (2020). Quantification of SARS-CoV-2 neutralizing antibody by a pseudotyped virus-based assay. *Nat Protoc* 15(11), 3699-3715. doi: 10.1038/s41596-020-0394-5.

Petrouskova, P., Bhide, K., Mochnacova, E., Kulkarni, A., Jozefiakova, J., Tkacova, Z., et al. (2025). Peptides developed against receptor binding sites of the E glycoprotein neutralize tick-borne encephalitis virus. *Sci Rep* 15(1), 11435. doi: 10.1038/s41598-025-95449-1.
